# A Self-Organizing State-Space-Model Approach for Parameter Estimation in Hodgkin-Huxley-Type Models of Single Neurons

**DOI:** 10.1371/journal.pcbi.1002401

**Published:** 2012-03-01

**Authors:** Dimitrios V. Vavoulis, Volko A. Straub, John A. D. Aston, Jianfeng Feng

**Affiliations:** 1 Department of Computer Science, University of Warwick, Coventry, United Kingdom; 2 Department of Cell Physiology and Pharmacology, University of Leicester, Leicester, United Kingdom; 3 Department of Statistics, University of Warwick, Coventry, United Kingdom; 4 Centre for Computational Systems Biology, Fudan University, Shanghai, PR China; Université Paris Descartes, Centre National de la Recherche Scientifique, France

## Abstract

Traditional approaches to the problem of parameter estimation in biophysical models of neurons and neural networks usually adopt a global search algorithm (for example, an evolutionary algorithm), often in combination with a local search method (such as gradient descent) in order to minimize the value of a cost function, which measures the discrepancy between various features of the available experimental data and model output. In this study, we approach the problem of parameter estimation in conductance-based models of single neurons from a different perspective. By adopting a hidden-dynamical-systems formalism, we expressed parameter estimation as an inference problem in these systems, which can then be tackled using a range of well-established statistical inference methods. The particular method we used was Kitagawa's self-organizing state-space model, which was applied on a number of Hodgkin-Huxley-type models using simulated or actual electrophysiological data. We showed that the algorithm can be used to estimate a large number of parameters, including maximal conductances, reversal potentials, kinetics of ionic currents, measurement and intrinsic noise, based on low-dimensional experimental data and sufficiently informative priors in the form of pre-defined constraints imposed on model parameters. The algorithm remained operational even when very noisy experimental data were used. Importantly, by combining the self-organizing state-space model with an adaptive sampling algorithm akin to the Covariance Matrix Adaptation Evolution Strategy, we achieved a significant reduction in the variance of parameter estimates. The algorithm did not require the explicit formulation of a cost function and it was straightforward to apply on compartmental models and multiple data sets. Overall, the proposed methodology is particularly suitable for resolving high-dimensional inference problems based on noisy electrophysiological data and, therefore, a potentially useful tool in the construction of biophysical neuron models.

## Author Summary

Parameter estimation is a problem of central importance and, perhaps, the most laborious task in biophysical modeling of neurons and neural networks. An emerging trend is to treat parameter estimation in this context as yet another statistical inference problem, which can be tackled using well-established methods from Computational Statistics. Inspired by these recent advances, we adopted a self-organizing state-space-model approach augmented with an adaptive sampling algorithm akin to the Covariance Matrix Adaptation Evolution Strategy in order to estimate a large number of parameters in a number of Hodgkin-Huxley-type models of single neurons. Parameter estimation was based on noisy electrophysiological data and involved the maximal conductances, reversal potentials, levels of noise and, unlike most mainstream work, the kinetics of ionic currents in the examined models. Our main conclusion was that parameters in complex, conductance-based neuron models can be inferred using the aforementioned methodology, if sufficiently informative priors regarding the unknown model parameters are available. Importantly, the use of an adaptive algorithm for sampling new parameter vectors significantly reduced the variance of parameter estimates. Flexibility and scalability are additional advantages of the proposed method, which is particularly suited to resolve high-dimensional inference problems.

## Introduction

Among several tools at the disposal of neuroscientists today, data-driven computational models have come to hold an eminent position for studying the electrical activity of single neurons and the significance of this activity for the operation of neural circuits [1]–[4]. Typically, these models depend on a large number of parameters, such as the maximal conductances and kinetics of gated ion channels. Estimating appropriate values for these parameters based on the available experimental data is an issue of central importance and, at the same time, the most laborious task in single-neuron and circuit modeling.

Ideally, all unknown parameters in a model should be determined directly from experimental data analysis. For example, based on a set of voltage-clamp recordings, the type, kinetics and maximal conductances of the voltage-gated ionic currents flowing through the cell membrane could be determined [5] and, then, combined in a conductance-based model, which replicates the activity of the biological neuron of interest under current-clamp conditions with sufficient accuracy. Unfortunately, this is not always possible, especially for complex compartmental models, which contain a large number of ionic currents.

A first problem arises from the fact that not all parameters can be estimated within an acceptable error margin, especially for small currents and large levels of noise. A second problem arises from the practice of estimating different sets of parameters based on data collected from different neurons of a particular type, instead of estimating all unknown parameters using data collected from a single neuron. Different neurons of the same type may have quite different compositions of ionic currents [6]–[9] (but, see also [10]). This implies that combining ionic currents measured from different neurons in the same model or even using the average of several parameters calculated over a population of neurons of the same type will not necessarily result in a model that expresses the experimentally recorded patterns of electrical activity under current-clamp conditions. Usually, only some parameters are well characterized, while others are difficult or impossible to measure directly. Thus, most modeling studies rely on a mixture of experimentally determined parameters and estimates of the remaining unknown ones using automated optimization methodology (see, for example, [11]–[22]). Typically, these methods require the construction of a cost function (for measuring the discrepancy between various features of the experimental data and the output of the model) and an automated parameter selection method, which iteratively generates new sets of parameters, such that the value of the cost function progressively decreases during the course of the simulation (see [23] for a review). Popular choices of such methods are evolutionary algorithms, simulated annealing and gradient descent methods. Often, a global search method (i.e. an evolutionary algorithm) is combined with local search (gradient descent) for locating multiple minima of the cost function with high precision. Since a poorly designed cost function (for example, one that merely matches model and experimental membrane potential trajectories) can seriously impede optimization, the construction of this function often requires particular attention (see, for example, [24]). Nevertheless, these computationally intensive methodologies have gained much popularity, particularly due to the availability of powerful personal computers at consumer-level prices and the development of specialized optimization software (e.g. [25]).

Alternative approaches also exist as, for example, methods based on the concept of synchronization between model dynamics and experimental data [26]. An emerging trend in parameter estimation methodologies for models in Computational Biology is to recast parameter estimation as an inference problem in hidden dynamical systems and then adopt standard Computational Statistics techniques to resolve it [27], [28]. For example, a particular study following this approach makes use of Sequential Monte Carlo methods (*particle filters*) embedded in an Expectation Maximization (EM) framework [28]. Given a set of electrophysiological recordings and a set of dynamic equations that govern the evolution of the hidden states, at each iteration of the algorithm the expected joint log-likelihood of the hidden states and the data is approximated using particle filters (Expectation Step). At a second stage during each iteration (Maximization Step), the log-likelihood is locally maximized with respect to the unknown parameters. The advantage of these methods, beyond the fact that they recast the estimation problem in a well-established statistical framework, is that they can handle various types of noisy biophysical data made available by recent advances in voltage and calcium imaging techniques.

Inspired by this emerging approach, we present a method for estimating a large number of parameters in Hodgkin-Huxley-type models of single neurons. The method is a version of Kitagawa's self-organizing state-space model [29] combined with an adaptive algorithm for selecting new sets of model parameters. The adaptive algorithm we have used is akin to the Covariance Matrix Adaption (CMA) Evolution Strategy [30], but other methods (e.g. Differential Evolution as described in [31]) may be used instead. We demonstrate the applicability of the algorithm on a range of models using simulated or actual electrophysiological data. We show that the algorithm can be used successfully with very noisy data and it is straightforward to apply on compartmental models and multiple datasets. An interesting result from this study is that by using the self-organizing state-space model in combination with a CMA-like algorithm, we managed to achieve a dramatic reduction in the variance of the inferred parameter values. Our main conclusion is that a large number of parameters in a conductance-based model of a neuron (including maximal conductances, reversal potentials and kinetics of gated ionic currents) can be inferred from low-dimensional experimental data (typically, a single or a few recordings of membrane potential activity) using the algorithm, if sufficiently informative priors are available, for example in the form of well-defined ranges of valid parameter values.

## Methods

### Modeling Framework

We begin by presenting the current conservation equation that describes the time evolution of the membrane potential for a single-compartment model neuron:
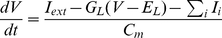
(1)where 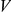, 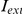 and 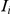 are all functions of time. In the above equation, 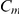 is the membrane capacitance, 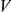 is the membrane potential, 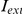 is the externally applied (injected) current, 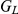 and 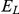 are the maximal conductance and reversal potential of the leakage current, respectively, and 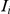 is the 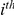 transmembrane ionic current. A voltage-gated current 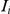 can be modeled according to the Hodgkin-Huxley formalism, as follows:
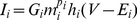
(2)where 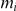 and 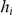 are both functions of time. In the above expression, 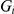 and 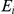 are the maximal conductance and reversal potential of the 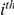 ionic current, 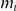 and 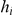 are dynamic gating variables, which model the voltage-dependent activation and inactivation of the current, and 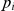 is a small positive integer power (usually, not taking values larger than 4). The product 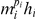 is the proportion of open channels in the membrane that carry the 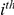 current. The gating variables 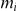 and 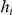 obey first-order relaxation kinetics, as shown below:
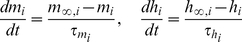
(3)where the steady states (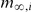, 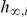) and relaxation times (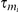, 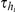) are all functions of voltage.

Using vector notation, we can write the above system of Ordinary Differential Equations (ODEs) in more concise form:
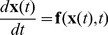
(4)where the state vector 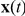 is composed of the time-evolving state variables 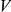, 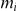 and 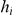 and the vector-valued function 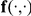, which describes the evolution of 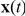 in time, is formed by the right-hand sides of Eqs. 1 and 3. Notice that 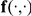 also depends on a parameter vector 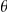, which for now is dropped from Eq. 4 for notational clarity. Components of 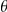 are the maximal conductances 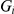, the reversal potentials 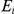 and the various parameters that control the voltage-dependence of the steady states and relaxation times in Eq. 3.

The above deterministic model does not capture the inherent variability in the electrical activity of neurons, but rather some average behavior of intrinsically stochastic events. In general, this variability originates from various sources, such as the random opening and shutting of transmembrane ion channels or the random bombardment of the neuron with external (e.g. synaptic) stimuli [32]. Here, we model the inherent variability in single-neuron activity by augmenting Eq. 4 with a noisy term and re-writing as follows:
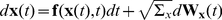
(5)where 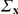 is a covariance matrix and 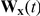 is a standard Wiener process over the state space of 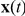. 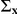 may be a diagonal matrix of variances (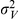, 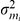 and 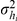) corresponding to each component of the state vector.

Typically, we assume that the above model is coupled to a measurement “device”, which permits indirect observations of the hidden state 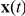:
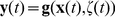
(6)where 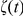 is an observation noise vector. In the simplest case, the vector of observations 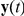 is one-dimensional and it may consist of noisy measurements of the membrane potential:
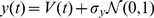
(7)where 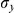 is the standard deviation of the observation noise and 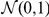 a random number sampled from a Gaussian distribution with zero mean and standard deviation equal to unity. More complicated non-linear, non-Gaussian observation functions may be used when, for example, the measurements are recordings of the intracellular calcium concentration, simultaneous recordings of the membrane potential and the intracellular calcium concentration or simultaneous recordings of the membrane potential from multiple sites (e.g. soma and dendrites) of a neuron.

Assuming that time 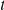 is partitioned in a very large number 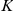 of time steps 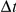, such that 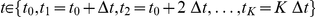 and the corresponding states are 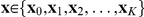, we can approximate the solution to Eq. 5 using the following difference equation:
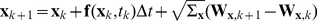
(8)where 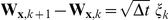 and 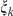 is a random vector with components sampled from a normal distribution with zero mean and unit variance. The above expression implements a simple rule for computing the membrane potential, activation and inactivation variables at each point 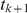 of the discretized time based on information at the previous time point 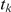 and it can be considered as a specific instantiation of the Euler-Maruyama method for the numerical solution of Stochastic Differential Equations [33].

Then, the observation model becomes:
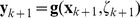
(9)In general, measurements do not take place at every point 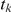 of the discretized time, but rather at intervals of 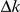 time steps (depending on the resolution of the measurement device), thus generating a total of 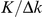 measurements. For simplicity in the above description, we have assumed that 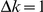. However, all the models we consider in the Results section assume 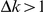.

In terms of probability density functions, the non-linear state-space model defined by Eqs. 8 and 9 (known as the *dynamics model*) and the *observation model*, respectively) can be written as:
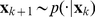
(10)

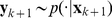
(11)where the initial state 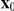 is distributed according to a prior density 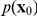. The above formulas are known as the *state transition* and *observation* densities, respectively [34].

### Simulation-Based Filtering and Smoothing

In many inference problems involving state-space models, a primary concern is the sequential estimation of the following two conditional probability densities [29]: (a) 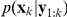 and (b) 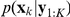, where 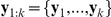, i.e. the set of observations (for example, a sequence of measurements of the membrane potential) up to the time point 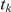. Density (a), known as the *filter* density, models the distribution of state 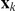 given all observations up to and including the time point 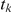, while density (b), known as the *smoother* density, models the distribution of state 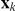 given the whole set of observations up to the final time point 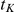.

In principle, the filter density can be estimated recursively at each time point 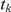 using Bayes' rule appropriately [29]:
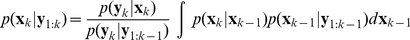
(12)where 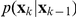 and 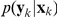 are the state transition and observation densities, respectively, and 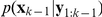 is the filter density at the previous time step 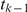.

Then, the smoother density can be obtained by using the following general recursive formula:
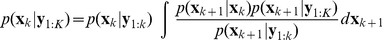
(13)which evolves backwards in time and makes use of the pre-calculated filter, 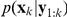. Given either of the above posterior densities, we can compute the expectation of any useful function of the hidden model state as:
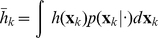
(14)where 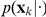 is either the filter or the smoother density. Common examples of 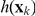 are 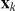 itself (giving the mean 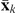) and the squared difference from the mean (giving the covariance of 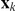).

In practice, the computations defined by the above formulas can be performed analytically only for linear Gaussian models using the Kalman smoother/filter and for finite state-space hidden Markov models. For non-linear models, the extended Kalman filter is a popular approach, which however can fail when non-Gaussian or multimodal density functions are involved [34]. A more generally applicable, albeit computationally more intensive approach, approximates the filter and smoother densities using Sequential Monte Carlo (SMC) methods, also known as *particle filters* [34], [35]. Within the SMC framework, the filter density at each time point is approximated by a large number 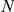 of discrete samples or *particles*, 
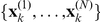
, and associated non-negative importance weights, 
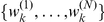
:
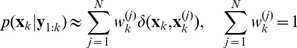
(15)where 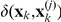 is the Dirac delta function centered at the 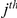 particle, 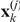.

Given an initial set of particles sampled from a prior distribution and their associated weights, a simple update rule involves the following steps [29]:

**Step 1:** For 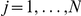, sample a new set of particles from the *proposal transition density function*, 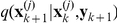. In general, one has enormous freedom in choosing the form of this density and even condition it on future observations, if these are available (see, for example, [36]). However, the simplest (and a quite common) choice is to use the transition density as the proposal, i.e. 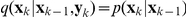. This is the approach we follow in this paper.

**Step 2:** For each new particle 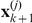, evaluate the importance weight:
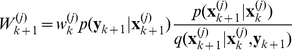
(16)Notice that when 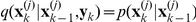, then the computation of the importance weights is significantly simplified, i.e. 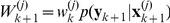.

**Step 3:** Normalize the computed importance weights, by dividing each of them with their sum, i.e.
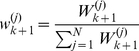
(17)The derived set of weighted samples 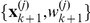 is considered an approximation of the filter density 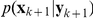.

In practice, the above algorithm is augmented with a re-sampling step (preceding Step 1), during which 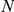 particles are sampled from the set of weighted particles computed at the previous iteration with probabilities proportional to their weights [34], [35]. All re-sampled particles are given weights equal to 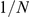. This step results in discarding particles with small weights and multiplying particles with large weights, thus compensating for the gradual degeneration of the particle filter i.e. the situation where all particles but one have weights equal to zero. For performance reasons, the resampling step may be applied only when the effective number of particles drops below a threshold value, e.g. 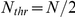. An estimation of the effective number of particles is given by
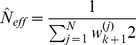
(18)


The above filter can be extended to a fixed-lag smoother, if instead of resampling just the particles at the current time step, we store and resample all particles up to 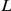 time steps before the current time step, i.e. 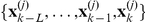 [29]. The resampled particles can be considered a realization from a posterior density 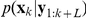, which is an approximation of the smoother density 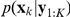, for sufficiently large values of 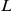.

Within this Monte Carlo framework, the expectation in Eq. 14 can be approximated as:
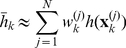
(19)for a large number 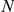 of weighted samples.

### Simultaneous Estimation of Hidden States and Parameters

It is possible to apply the above standard filtering and smoothing techniques to parameter estimation problems involving state-space models. The key idea [29] is to define an extended state vector 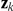 by augmenting the state vector 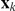 with the model parameters, i.e. 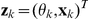. Then, the time evolution of the extended state-space model becomes:
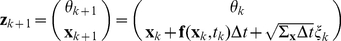
(20)while the observational model remains unaltered:
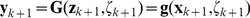
(21)The marginal posterior density of the parameter vector 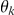 is given by:
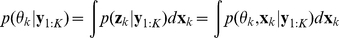
(22)and, subsequently, the expectation of any function of 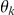 can be computed as in Eq. 14:
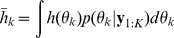
(23)Furthermore, given a set of particles and associated weights, which approximate the smoother density 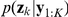 as outlined in the previous section, i.e. 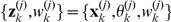 for 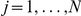, the above expectation can be approximated as:
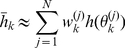
(24)for large 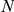.

Under this formulation, parameter estimation, which is traditionally treated as an optimization problem, is reduced to an integration problem, which can be tackled using filtering and smoothing methodologies for state-space models, a well-studied subject in the field of Computational Statistics.

### Connection to Evolutionary Algorithms

It should be emphasized that although in Eq. 20 the parameter vector 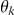 was assumed constant, i.e. 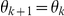, the same methodology applies in the case of parameters that are naturally evolving in time, such as a time-varying externally injected current 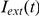. A particularly interesting case arises when an artificial evolution rule is imposed on a parameter vector, which is otherwise constant by definition. Such a rule allows sampling new parameter vectors based on samples at the previous time step, i.e. 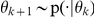, and generating a sequence 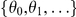, which explores the parameter space and, ideally converges in a small optimal subset of it, after a sufficiently large number of iterations. It is at this point that the opportunity to use techniques borrowed from the domain of Evolutionary Algorithms arises. Here, we assume that the artificial evolution of the parameter vector 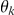 is governed by a version of the Covariance Matrix Adaptation algorithm [30], a well-known Evolution Strategy, although the modeler is free to make other choices (e.g. Differential Evolution [31]). For the 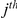 particle, we write:
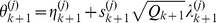
(25)where 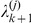 is a random vector with elements sampled from a normal distribution with zero mean and unit variance. 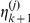 and 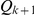 are a mean vector and covariance matrix respectively, which are computed as follows:
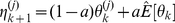
(26)

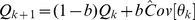
(27)In the above expressions, a and 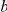 are small adaptation constants and 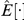 and 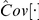 are the expectation and covariance of the weighted sample of 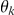, respectively. 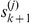 is a scale parameter that evolves according to a log-normal update rule:
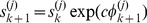
(28)where 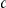 is a small adaptation constant and 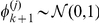 is a normally distributed random number with zero mean and unit variance.

According to Eq. 25, the parameter vector 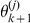 is sampled at each iteration of the algorithm from a multivariate normal distribution, which is centered at 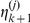 and has a covariance matrix equal to 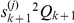:
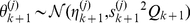
(29)Both 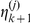 and 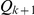 are slowly adapting to the sample mean 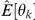 and covariance 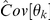, with an adaptation rate determined by the constants 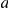 and 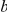. Notice that by switching off the adaptation process (i.e. by setting 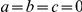), 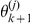 evolves according to a multivariate Gaussian distribution, which is centered at the previous parameter vector and has a covariance matrix equal to 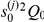:
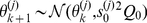
(30)Therefore, given an initial set of weighted particles 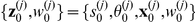 sampled from some prior density function and an initial covariance matrix 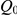, which may be set equal to the identity matrix, the smoothing algorithm presented earlier becomes:

**Step 1a:** Compute the expectation 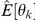 and covariance 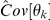 of the weighted sample of 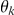


**Step 1b:** For 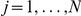, compute the scale factor 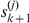 according to Eq. 28. Notice that this scale factor is now part of the extended state 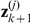 for each particle

**Step 1c:** For 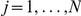, compute the mean vector 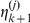, as shown in Eq. 26

**Step 1d:** Compute the covariance matrix 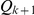, as shown in Eq. 27

**Step 1e:** For 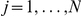, sample 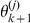, as shown in Eq. 25

**Step 1f:** For 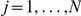, sample a new set of state vectors from the proposal density 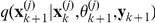, thus completing sampling the extended vectors 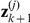. Notice that the proposal density 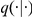 is conditioned on the updated parameter vector 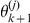.

**Step 2–3:** Execute steps 2 and 3 as described previously

Notice that in the algorithm outlined above, the order in which the components of 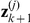 are sampled is important. First, we sample the scaling factor 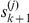. Then, we sample the parameter vector 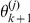 given the updated 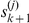. Finally, we sample the state vector 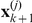 from a proposal, which is conditioned on the updated parameter vector 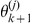. When resampling occurs, the state vectors 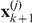 with large importance weights are selected and multiplied with high probability along with their associated parameter vectors and scaling factors, thus resulting in a gradual self-adaptation process. This self-adaptation mechanism is very common in the Evolution Strategies literature.

### Implementation

The algorithm described in the previous section was implemented in MATLAB and C (source code available as Supplementary Material; unmaintained FORTRAN code is also available upon request from the first author) and tested on parameter inference problems using simulated or actual electrophysiological data and a number of Hodgkin-Huxley-type models: (a) a single-compartment model (derived from the classic Hodgkin-Huxley model of neural excitability) containing a leakage, transient sodium and delayed rectifier potassium current, (b) a two-compartment model of a cat spinal motoneuron [37] and (c) a model of a B4 motoneuron in the Central Nervous System of the pond snail *Lymnaea stagnalis* [38], which was developed as part of this study. Each of these models is described in detail in the Results section. Models (a) and (b) were used for generating noisy voltage traces at a sampling rate of 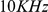 (one sample every 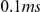). The simulated data was subsequently used as input to the algorithm in order to estimate a large number of parameters; typically, maximal conductances of ionic currents, reversal potentials, the parameters governing the activation and inactivation kinetics of ionic currents, as well as the levels of intrinsic and observation noise. Estimated parameter values were subsequently compared against the true parameter values in the model. The MATLAB environment was used for visualization and analysis of simulation results. For the estimation of the unknown parameters in model (c), actual electrophysiological data were used, as described in the next section.

Prior information was incorporated in the smoother by assuming that parameter values were not allowed to exceed well-defined upper or lower limits (see Tables 1, 2 and 3). For example, maximal conductances never received negative values, while time constants were always larger than zero. At the beginning of each simulation, the initial population of particles was uniformly sampled from within the acceptable range of parameter values and, during each simulation, parameters were forced to remain within their pre-defined limits.

**Table 1:** True and estimated values and prior intervals used during smoothing for all parameters in the single-compartment conductance-based model.

#	Parameter	Unit	True Value	Estimated Value^1^	Lower Bound	Upper Bound
1	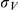	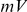	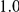	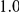	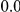	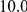
2	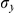	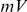	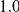	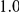	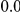 1	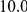
3	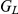	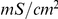	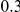	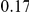	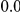	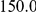
4	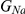	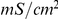	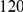 .0	34.3	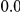	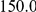
5	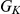	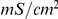	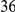 .0	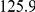	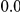	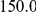
6	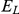	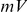	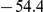	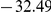	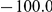	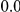
7	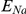	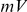	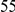 .0	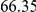	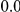	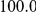
8	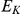	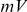	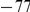 .0	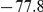	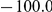	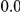
9	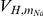	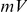	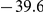	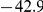	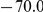 **(−45.0)** 2	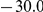 **(−35.0)**
10	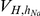	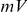	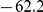	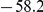	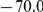 **(−65.0)**	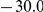 **(−55.0)**
11	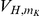	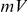	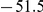	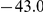	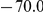 **(−55.0)**	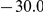 **(−45.0)**
12	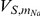	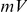	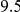	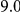	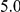 **(5.0)**	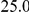 **(10.0)**
13	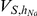	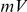	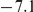	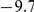	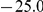 **(−10.0)**	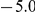 **(−5.0)**
14	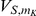	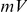	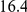	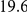	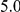 **(10.0)**	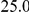 **(20.0)**
15	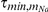	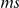	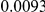	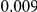	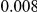	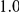
16	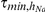	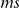	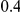	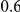	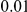	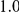
17	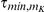	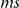	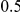	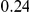	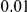	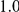
18	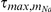	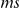	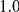	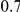	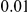	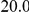
19	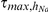	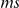	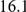	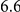	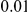	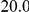
20	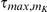	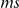	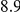	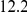	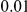	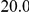
21	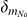	-	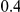	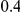	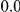 **(0.0)**	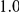 **(0.5)**
22	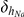	-	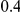	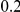	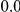 **(0.0)**	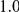 **(0.5)**
23	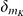	-	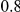	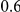	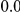 **(0.5)**	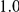 **(1.0)**

1 These parameter values were estimated when we used the broad prior intervals (see Fig. 7Di).

2 Values in bold indicate the narrow prior intervals we used for generating Fig. 7Dii (and Supplementary Fig. S3).

**Table 2:** True and estimated values and prior intervals used during smoothing for all parameters in the two-compartment conductance-based model.

#	Parameter	Unit	True Value	Estimated Value^1^	Lower Bound	Upper Bound
1	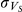	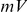	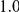	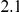	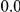	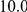
2	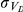	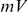	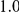	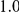	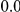	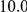
3	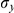	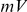	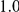	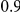	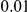	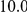
4	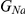	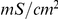	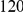	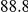	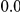	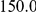
5	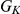	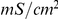	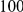	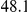	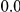	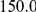
6	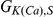	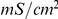	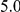	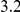	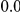	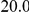
7	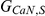	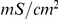	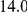	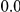	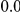	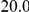
8	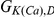	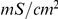	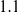	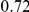	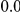	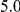
9	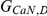	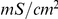	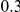	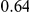	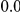	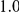
10	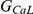	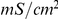	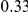	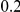	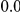	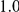
11	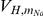	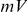	−35.0	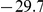	−60.0 **(−45.0)** 2	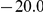 **(−25.0)**
12	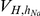	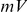	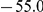	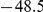	−60.0 **(−65.0)**	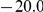 **(−45.0)**
13	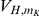	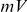	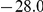	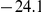	−60.0 **(−40.0)**	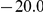 **(−20.0)**
14	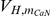	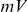	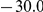	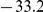	−60.0 **(−40.0)**	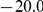 **(−20.0)**
15	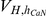	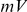	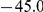	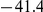	−60.0 **(−55.0)**	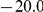 **(−35.0)**
16	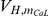	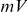	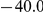	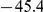	−60.0 **(−50.0)**	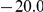 **(−30.0)**
17	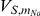	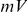	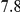	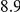	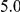 **(5.0)**	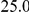 **(10.0)**
18	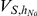	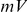	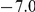	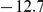	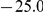 **(−10.0)**	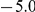 **(−5.0)**
19	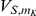	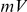	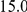	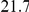	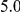 **(10.0)**	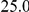 **(20.0)**
20	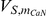	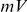	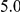	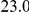	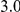 **(3.0)**	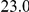 **(8.0)**
21	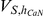	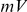	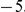 0	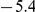	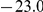 **(−8.0)**	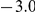 **(−3.0)**
22	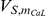	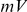	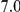	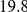	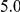 **(5.0)**	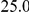 **(10.0)**
23	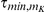	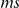	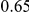	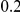	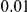	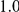
24	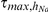	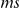	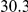	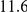	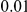	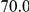
25	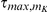	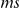	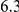	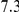	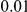	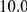
26	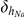	-	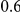	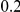	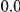 **(0.5)**	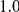 **(1.0)**
27	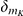	-	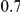	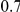	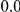 **(0.5)**	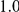 **(1.0)**
28	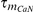	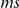	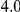	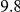	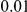	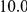
29	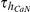	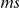	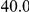	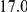	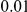	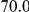
30	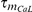	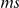	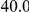	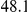	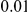	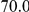

1 These parameter values were estimated when we used the broad prior intervals (see Fig. 11Ai).

2 Values in bold indicate the narrow prior intervals we used for generating Figs. 11Aii, 11B, 11C (and Supplementary Figs. S4 and S5).

**Table 3:** Estimated mean values and prior limits used during smoothing for all parameters in the B4 model.

#	Parameter	Unit	Estimated Mean Value^1^^,^ 2	Lower Bound	Upper Bound
1	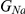	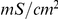	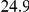	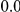	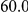
2	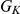	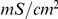	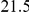	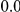	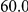
3	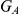	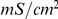	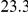	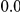	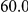
4	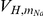	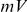	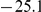	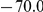 **(−40.0)**	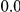 **(−20.0)**
5	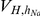	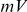	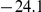	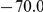 **(−40.0)**	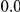 **(−20.0)**
6	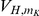	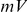	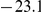	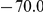 **(−40.0)**	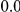 **(−20.0)**
7	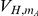	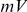	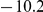	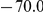 **(−20.0)**	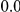 **(0.0)**
8	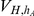	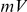	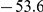	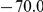 **(−70.0)**	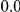 **(−40.0)**
9	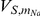	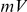	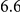	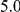 **(5.0)**	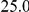 **(10.0)**
10	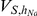	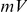	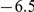	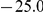 **(−10.0)**	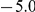 **(−5.0)**
11	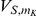	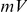	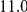	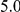 **(10.0)**	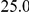 **(15.0)**
12	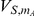	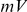	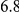	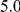 **(5.0)**	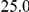 **(10.0)**
13	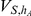	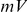	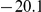	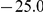 **(−25.0)**	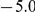 **(−15.0)**
14	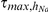	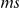	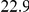	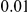 **(15.0)**	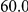 **(25.0)**
15	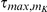	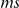	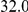	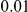 **(25.0)**	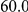 **(35.0)**
16	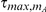	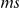	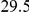	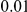 **(25.0)**	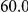 **(35.0)**
17	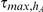	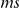	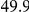	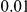 **(35.0)**	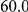 **(60.0)**

1 These parameter values were estimated when we used the narrow prior intervals (in bold; see Fig. 12).

2 The parameter posteriors estimated when we used the broad prior intervals are illustrated in Supplementary Fig. S7.

All simulations were performed on an Intel dual-core i5 processor with 4 GB of memory running Ubuntu Linux. The number of particles used in each simulation was typically 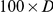, where 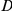 was the dimensionality of the extended state 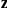 (equal to the number of free parameters and dynamic states in the model). The time step 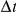 in the Euler-Maruyama method was set equal to 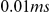. The parameter 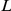 of the fixed-lag smoother was set equal to 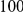 (unless stated otherwise), which is equivalent to a time window 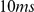 wide (since data were sampled every 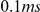). The adaptation constants 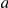, 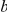 and 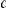 in Eqs. 26, 27 and 28 were all set equal to 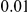, unless stated otherwise. Depending on the size of 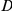, the complexity of the model and the length of the (actual or simulated) electrophysiological recordings, simulation times ranged from a few minutes up to more than 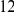 hours.

### Electrophysiology

As part of this study, we developed a single-compartment Hodgkin-Huxley-type model of a B4 neuron in the pond-snail *Lymnaea stagnalis* [38]. B4 neurons are part of the neural circuit that controls the rhythmic movements of the feeding muscles via which the animal captures and ingests its food. The *Lymnaea* central nervous system was dissected from adult animals (shell length 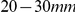) that were bred at the University of Leicester as described previously [39]. All dissections were carried out in 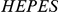-buffered saline containing (in 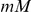) 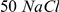, 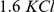, 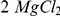, 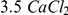, and 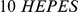, 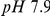, in distilled water. All chemicals were purchased from Sigma. The buccal ganglia containing the B4 neurons were separated from the rest of the nervous system by cutting the cerebral buccal connectives and the buccal-buccal connective was crushed to eliminate electrical coupling between B4 neurons in the left and right buccal ganglion. Prior to recording, excess saline was removed from the dish and small crystals of protease type XIV were placed directly on top of the buccal ganglia to soften the connective tissue and aid the impalement of individual neurons. The protease crystals were washed of after about 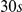 with multiple changes of 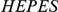-buffered saline. The B4 neuron was visually identified based on its size and position and impaled with two sharp intracellular electrodes filled with a mixture of 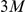 potassium acetate and 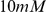 potassium chloride (resistance 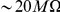). During the recording, the preparation was bathed in 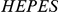-buffered saline plus 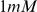 hexamethonium chloride to block cholinergic synaptic inputs and suppress spontaneous fictive feeding activity.

The signals from the two intracellular electrodes were amplified using a Multiclamp 900A amplifier (Molecular Devices), digitized at a sampling frequency of 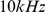 using a CED1401plus A/D converter (Cambridge Electronic Devices) and recorded on a PC using Spike2 version 6 software (Cambridge Electronic Devices). A custom set of instructions using the Spike2 scripting language was used to generate sequences of current pulses consisting of individual random steps ranging in amplitude from 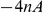 to 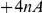 and a duration from 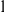 to 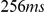. The current signal was injected through one of the recording electrodes whilst the second electrode was used to measure the resulting changes in membrane potential.

## Results

### Hidden States, Intrinsic and Observational Noise are Simultaneously Estimated Using the Fixed-Lag Smoother

The applicability of the fixed-lag smoother presented above was demonstrated on a range of Hodgkin-Huxley-type models using simulated or actual electrophysiological data. The first model we examined consisted of a single compartment containing leakage, sodium and potassium currents, as shown below:
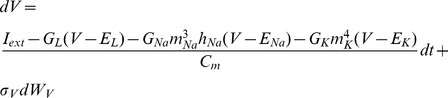


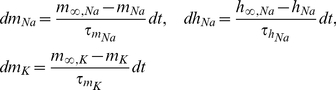
(32)where 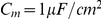. Notice the absence of noise in the dynamics of 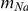, 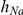 and 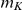, which is valid if we assume a very large number of channels (see Supplementary Material and Supplementary Figures S1 and S2 for the case were noise is present in the dynamics of these variables). The steady states and relaxation times of the activation and inactivation gating variables were voltage-dependent, as shown below (e.g. [5]):
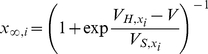
(33)and
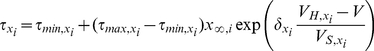
(34)where 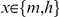 and 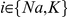. The parameters 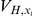, 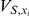, 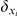, 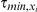 and 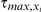 in Eqs. 33 and 34 were chosen such that 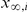 and 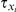 fit closely the corresponding steady-states and relaxation times of the classic Hodgkin-Huxley model of neural excitability in the giant squid axon [40]. Observations consisted of noisy measurements of the membrane potential, as shown in Eq. 7. The full set of parameter values in the above model is given in Table 1.

**Figure 1:**
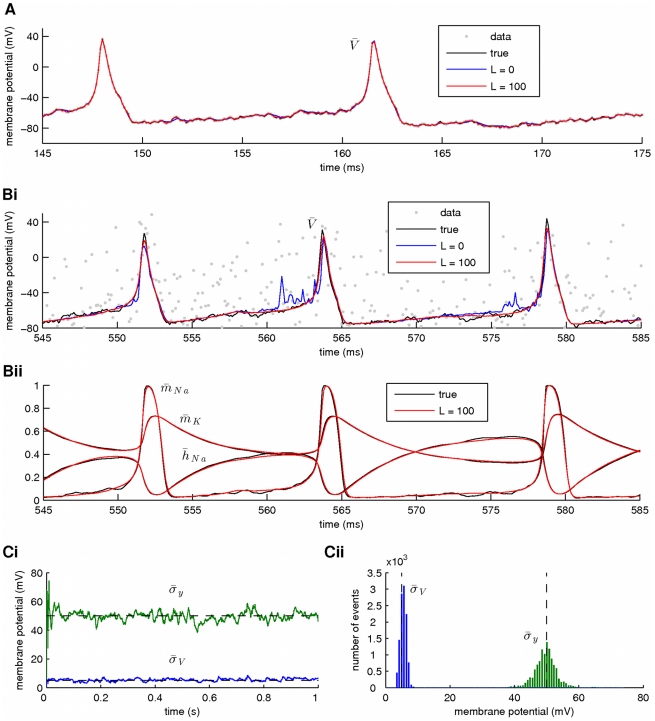
Simultaneous estimation of hidden states, intrinsic and observation noise. Estimation was based on a simulated recording of membrane potential with duration 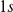. For clarity, only 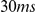 of activity are shown in A and Bi,ii. (**A**) Smoothing of the membrane potential (the observed variable), when observation noise was low (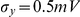). High-fidelity smoothing was achieved for either small (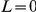) or large (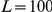) values of the fixed smoothing lag 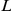. Simulated and smoothed data are difficult to distinguish due to their overlap. (**Bi**) Smoothing of the membrane potential at high levels of observation noise (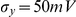). A large value of the smoothing lag (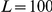) was required for high-fidelity smoothing. (**Bii**) Inference of the unobserved activation (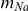, 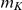) and inactivation (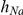) variables for sodium and potassium currents as functions of time, during smoothing of the data shown in Bi for 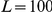. (**Ci**) Inference of the standard deviations for the intrinsic and observation noise (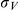 and 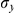, respectively) during smoothing of the data shown in Bi for 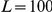. Dashed lines indicate the true values of 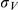 and 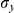. (**Cii**) Histograms of the time series for 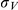 and 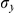 in Ci. Again, dashed lines indicate the true values of the corresponding parameters. At this stage, maximal conductances, reversal potentials and kinetic parameters in the model were assumed known. The number of particles was 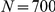. Also, 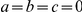. The scaling factors in Eq. 25 were all considered equal to 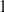.

First, we used the fixed-lag smoother to simultaneously infer the hidden states (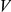, 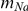, 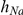, 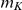) and standard deviations of the intrinsic (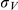) and observation (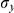) noise based on 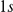-long simulated recordings of the membrane potential 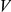. These recordings were generated by assuming a time-dependent 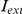 in Eq. 31, which consisted of a sequence of current steps with amplitude randomly distributed between 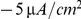 and 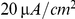 and random duration up to a maximum of 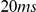. Two simulated voltage recordings were generated corresponding to two different levels of observation noise, 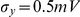 and 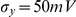, respectively. The second value (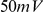) was rather extreme and it was chosen in order to illustrate the applicability of the method even at very high levels of observation noise. Simulated data points were sampled every 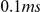 (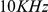). The standard deviation of the intrinsic noise was set at 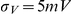. The injected current 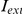 and the induced voltage trace (for either value of 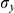) were then used as input to the smoother, during the inference phase. At this stage, all other parameters in the model (conductances, reversal potentials, and ionic current kinetics) were assumed known, thus the extended state vector took the form 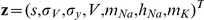, where 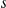 was a scale factor as in Eq. 25. New samples for 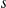 were taken from a log-normal distribution (Eq. 28), while new samples for 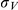 and 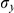 were drawn from an adaptive bivariate Gaussian distribution at each iteration of the algorithm (Eq. 25). For each data set, smoothing was repeated for two different values of the smoothing lag, i.e. 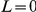 and 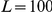. 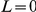 corresponds to filtering, while 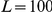 corresponds to smoothing with a fixed lag equal to 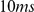. Our results from this set of simulations are summarized in Fig. 1.

We observed that at low levels of observation noise (Fig. 1A), the inferred expectation of the voltage (solid blue and red lines) closely matched the underlying (true) signal (solid black line). This was true for both values of the fixed lag 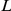 used for smoothing. However, at high levels of observation noise (Fig. 1Bi), the true voltage was inferred with high fidelity when a large value of the fixed lag (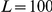) was used (solid red line), but not when 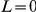 (solid blue line). Furthermore, the inferred expectations of the unobserved dynamic variables 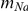, 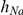 and 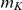 (solid red lines in Fig. 1Bii) also matched the true hidden time series (solid black lines in the same figure) remarkably well, when 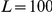.

We repeat that during these simulations an artificial update rule was imposed on the two free standard deviations 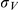 and 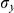, as shown in Eq. 25. The artificial evolution of these parameters is illustrated in Fig. 1Ci, where the inferred expectations of 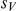 and 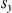 are presented as functions of time. These expectations converged immediately, fluctuating around the true values of 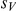 and 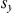 (dashed lines in Fig. 1Ci). This is also illustrated by the histograms in Fig. 1Cii, which were constructed from the data points in Fig. 1Ci. We observed that the peaks of these histograms were located quite closely to the true values of 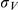 and 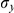 (dashed lines in Fig. 1Cii).

In summary, the fixed-lag smoother was able to recover the hidden states and standard deviations of the intrinsic and observation noise in the model based on noisy observations of the membrane potential. This was true even at high levels of observation noise, subject to the condition that a sufficiently large smoothing lag 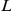 was adopted during the simulation.

### Adaptive Sampling Reduces the Variance of Inferred Parameter Distributions and Accelerates Convergence of the Algorithm

Next, we treated two more parameters in the model as unknown, i.e. the maximal conductances of the transient sodium (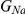) and delayed rectifier potassium (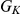) currents. The extended state vector, thus, took the form 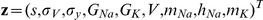. As in the previous section, new samples for 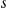 were drawn from a log-normal distribution (Eq. 28), while 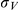, 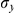, 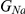 and 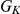 were sampled by default from an adaptive multivariate Gaussian distribution at each iteration of the algorithm (Eq. 25).

**Figure 2:**
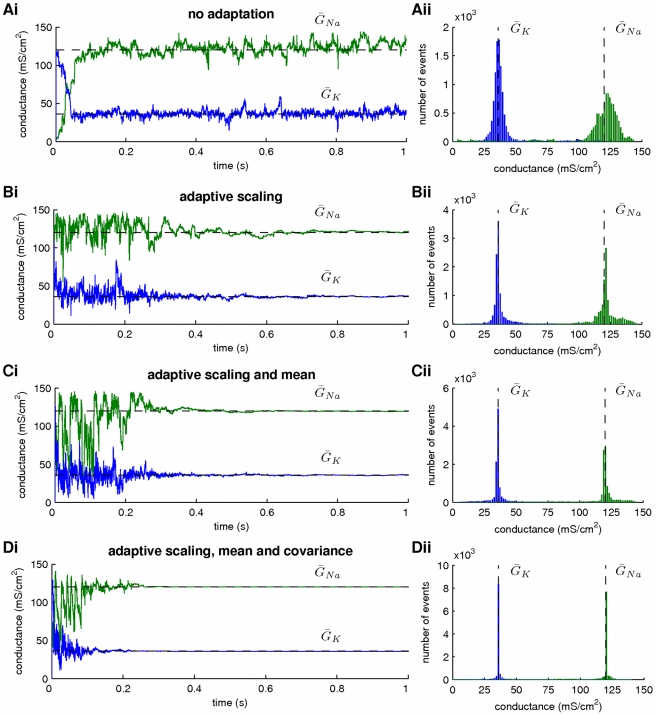
The effect of adaptive parameter sampling on the variance of parameter estimates. Merging the fixed-lag smoother with an adaptive sampling algorithm akin to the Covariance Matrix Adaptation Evolution Strategy reduced significantly the variance of parameter estimates. At this stage, the maximal conductances for the sodium (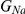) and potassium (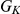) currents were assumed unknown. Estimation was based on a simulated recording of membrane potential with duration 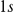 and 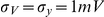. (**A**) Inference of 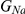 and 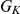 during smoothing, when new parameter samples were drawn from a non-adaptive multi-variate normal distribution (Eq. 30). Dashed lines indicate the true parameter values. (**B**) Inference of 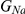 and 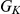 during smoothing, when new samples were drawn from a multi-variate normal distribution (Eq. 25) with an adaptive scaling factor 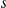 (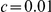 in Eq. 28). (**C**) Inference of 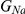 and 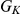 during smoothing, when new samples were drawn from a multi-variate normal distribution (Eq. 25) with adaptive scaling (as in B) and mean (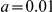 in Eq. 26). (**D**) Inference of 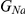 and 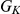 during smoothing, when new samples were drawn from a multi-variate normal distribution with adaptive scaling (as in B), mean (as in C) and covariance (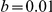 in Eq. 27). The histograms in the right plots were constructed from the time series in the left plots. Membrane potential, activation and inactivation variables, intrinsic and observation noise were also subject to estimation, as in Fig. 1. Smoothing lag and number of particles were 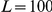 and 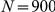, respectively. The prior interval of the scaling factors 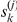 was 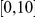.

In order to examine the effect of this adaptive sampling approach on the variance of the inferred parameter distributions, we repeated fixed-lag smoothing on 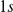-long simulated recordings of the membrane potential assuming each time that different aspects of the adaptive sampling process were switched off, as illustrated in Fig. 2. First, we assumed that no adaptation was imposed on 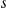 or the “unknown” noise parameters and maximal conductances, i.e. the constants 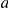, 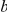 and 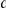 in Eqs. 26–28 were all set equal to zero. In this case, the multivariate Gaussian distribution from which new samples of 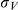, 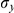, 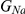 and 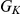 were drawn from reduced to Eq. 30. In addition, we assumed that 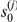 in the same equation was equal to 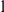, for all samples 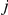. Under these conditions, the true values of the free parameters were correctly estimated through application of the fixed-lag smoother, as illustrated for the case of 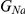 and 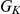 in Figs. 2Ai and 2Aii.

Subsequently, we repeated smoothing assuming that the scale factor 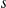 evolved according to the log-normal update rule given by Eq. 28 with 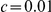, while 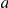 and 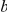 were again set equal to 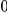. As illustrated in Figs. 2Bi and 2Bii for parameters 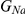 and 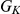, by imposing this simple adaptation rule on the multivariate Gaussian distribution from which the free parameters in the model were sampled, we managed again to estimate correctly their values, but this time the variance of the inferred parameter distributions (the width of the histograms in Fig. 2Bii) was drastically reduced.

By further letting the mean and covariance of the proposal Gaussian distribution in Eq. 25 adapt (by setting 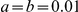 in Eqs. 26 and 27), we achieved a further decrease in the spread of the inferred parameter distributions (Figs. 2C and 2D). Parameters 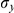 and 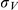 and the hidden states 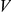, 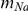, 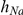 and 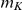 were also inferred with very high fidelity in all cases (as in Fig. 1), but the variance of the estimated posteriors for 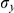 and 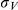 followed the same pattern as the variance of 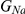 and 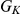.

It is worth observing that when all three adaptation processes were switched on (i.e. 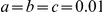), the algorithm converged to a single point in parameter space within the first 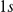 of simulation, which coincided with the true parameter values in the model (see Fig. 2D for the case of 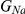 and 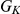). At this point, the covariance matrix 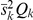 became very small (i.e. all its elements were less than 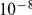, although the matrix itself remained non-singular) and the mean 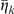 was very close to the true parameter vector 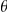. We note that 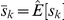 and 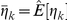, where 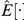 stands for the expectation computed over the population of particles. In this case, it is not strictly correct to claim that the chains in Fig. 2Di approximate the posteriors of the unknown parameters 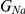 and 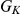; since repeating the simulation many times would result in convergence at slightly different points clustered tightly around the true parameter values, it would be more reasonable to claim that these optimal points are random samples from the posterior parameter distribution and they can be treated as estimates of its mode.

**Figure 3:**
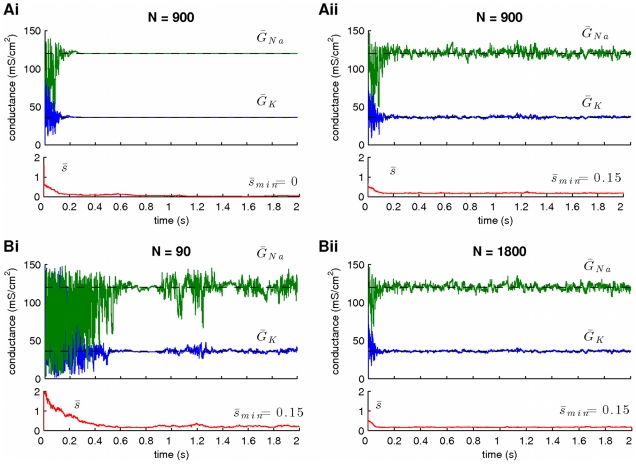
The effect of the size of the scaling factor 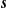
 **and the number of particles** 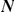 **on the variance of the estimates.** Large minimal values of 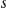 and small values of 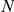 imply large variance of the estimates. (**A**) Resampling of particles (see Methods) implies adaptation of (among others) the scaling factors 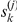, which gradually approach the lower bound of their prior interval (red lines in Ai,ii). A prior interval with zero lower bound (i.e. 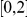) leads to estimates with negligible variance (Ai). A prior interval with relatively large lower bound (e.g. 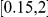) leads to estimates with non-zero variance (Aii). Notice that the expectation 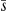 in Ai does not actually take the value 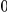 (instead it becomes approximately equal to 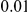). (**B**) A small number of particles (Bi, 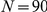) implies estimates with large variance (compare to Bii, 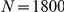). Notice that the difference between Aii (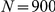) and Bii (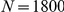) is negligible, implying the presence of a ceiling effect, when the number of particles becomes very large. In these simulations, 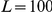 and 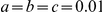.

Depending on the situation, one may wish to estimate the full posteriors of the unknown parameters or just an optimal set of parameter values, which can be used in a subsequent predictive simulation. In Fig. 3A, we examined in more detail how the scale factor 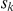 affects the variance of the final estimates, assuming that 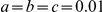. We repeat that each particle 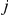 contains 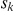 as a component of its extended state. Each scaling factor 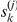 is updated at each iteration of the algorithm following a lognormal rule (Eq. 28, Step 1b of the algorithm in the Methods section). Sampling new parameter vectors is conditioned on these updated scaling factors (Eq. 25, Step 1e of the algorithm). When at a later stage weighting (and resampling) of the particles occurs, the scaling factors that are associated with high-weight parameters and hidden states are likely to survive into subsequent iterations (or “generations”) of the algorithm. During the course of this adaptive process, the scaling factors 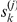 are allowed to fluctuate only within predefines limits, similarly to the other components of the extended state vector.

In Fig. 3Ai, we demonstrate the case where the scaling factors 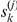 were allowed to take values from the prior interval 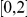. We observed that during the course of the simulation (which utilized 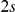-long simulated membrane potential recordings), the average value of the scaling factor, 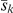, decreased gradually towards 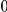 and this was accompanied by a dramatic decrease in the variance of the inferred parameters 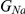 and 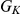, which eventually “collapsed” to a point in parameter space located very close to their true values. This situation was the same as the one illustrated in Fig. 2D. Notice that although 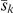 decreased towards zero, it never actually took this value; it merely became very small (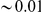). When we used a prior interval for 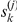 with non-zero lower bound (i.e. 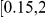]; see Fig. 3Aii), the final estimates had a larger variance, providing an approximation of the full posteriors of the “unknown” parameters 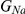 and 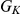. Thus, controlling the lower bound of the prior interval for the scaling factors 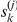 provides a simple method for controlling the variance of the final estimates. Notice that the variance of the final estimates also depends on the number of particles (Fig. 3B). A smaller number of particles resulted in a larger variance of the estimates (compare Fig. 3Bi to Fig. 3Bii). However, when a large number of particles was already in use, further increasing their number did not significantly affect the variance of the estimates or the rate of convergence (compare Fig. 3Bii to Fig. 3Aii), indicating the presence of a ceiling effect.

**Figure 4:**
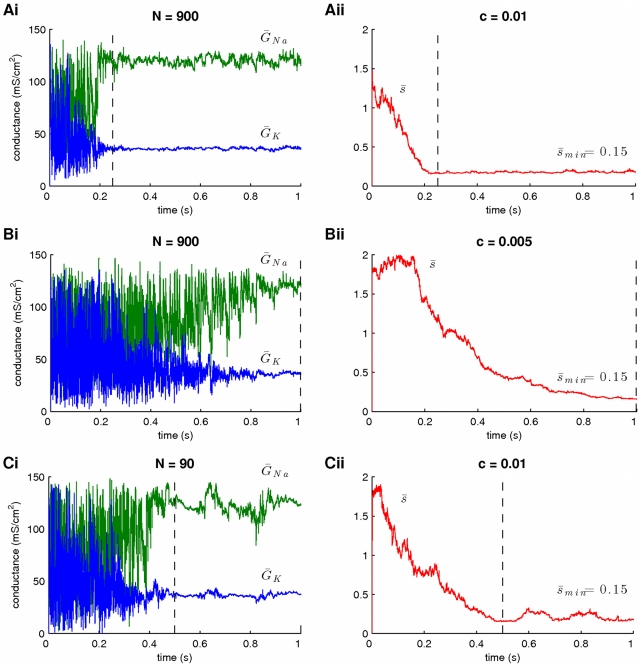
The effect of adaptation of the scaling factor 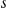
 **and the number of particles** 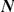 **on the speed of convergence.** A slow rate of adaptation for 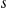 and a small number of particles 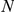 imply slow convergence of the algorithm. The rate at which 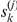 adapts depends on the parameter 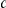 in Eq. 28. Reducing 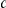 in half results in a significant decrease in the rate of convergence (compare A to B). Also, reducing the number of particles by a factor of 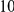 slows down the speed of convergence (compare A to C), but not as much as when parameter 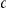 was adjusted. The plots on the right illustrate the profile of 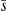 associated with the estimation of the parameters on the left plots. In these simulations, 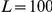, 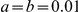 and the prior interval for the scaling factors 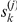 was 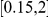.

The adaptive sampling of the scaling factors 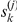 further depends on parameter 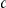 in Eq. 28, which determines the width of the lognormal distribution from which new samples are drawn. The value of this parameter provides a simple way to control the rate of convergence of the algorithm; larger values of 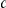 resulted in faster convergence, when processing 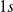-long simulated recordings (compare Fig. 4A to Fig. 4B). The rate of convergence also depends on the number of particles in use (compare Fig. 4A to Fig. 4C), although it is more sensitive to changes in parameter 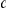; dividing the value of 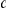 by 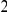 (Fig. 4B) had a larger effect on the rate of convergence than dividing the number of particles by 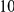 (Fig. 4C).

In summary, by assuming an adaptive sampling process for the unknown parameters in the model, we managed to achieve a significant reduction in the spread of the inferred posterior distributions of these parameters. Furthermore, adjusting the prior interval and adaptation rate 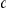 of the scaling factors 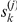 provides a straightforward way to control the variance of the estimated posteriors and the rate of convergence of the algorithm. Alternatively, we could have set 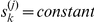, i.e. set it to the same constant value for all particles 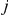 and time steps 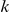 (as in Fig. 2A). However, by permitting 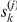 to adapt within a predefined interval, we potentially allow this parameter and, thus, the covariance matrices 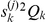 take large values, which in turn would permit the algorithm to escape local optima in the parameter space. For example, the time profiles of 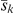 in Figs. 3 and 4 indicate that, early during the simulations, this quantity had relatively large values, which were associated with large variances of the posterior parameter estimates. During this initial period, the algorithm has the potential to “jump” away from local optima and towards more optimal regions of the parameter space. One may see, here, a distant analogy to simulated annealing, where a fictitious “temperature” control variable is gradually decreased, thus allowing the system to escape local minima and gradually settle to more optimal regions of the energy landscape.

### Increasing Observation Noise Reduces the Accuracy and Precision of the Fixed-Lag Smoother

In a subsequent stage, we treated as unknown two more parameters in the model, i.e. the reversal potentials for the sodium and potassium currents, 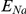 and 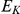, respectively. Thus, the extended state vector became 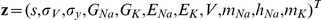. This time, we wanted to examine how increasing levels of observation noise (i.e. the value of parameter 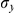) affect the inference of unknown quantities in the model based on the fixed-lag smoother. For this reason, we repeated smoothing on four 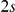-long simulated data sets (i.e. recordings of membrane potential and the associated 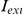) corresponding to increasing values of the standard deviation of the observation noise 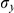, i.e. 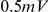, 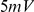, 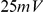 and 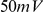.

**Figure 5:**
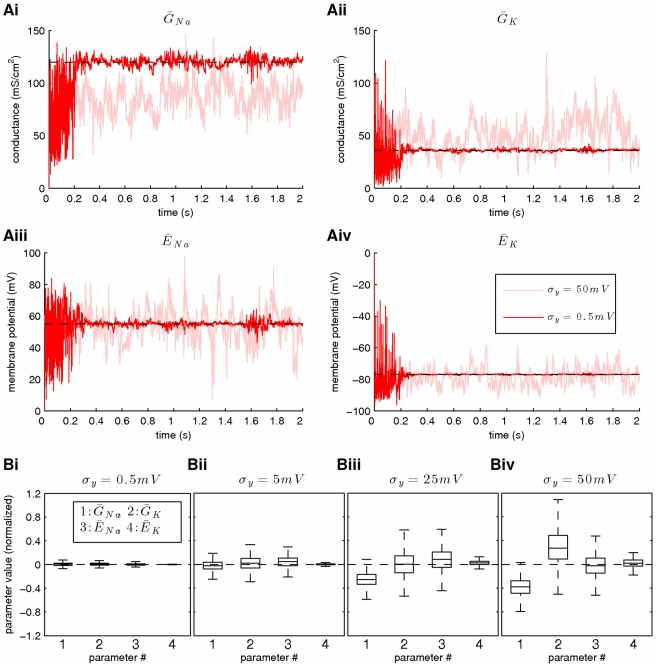
The effect of observation noise on the accuracy and precision of parameter estimates. Increasing observation noise decreases the accuracy and precision of the fixed-lag smoother. At this stage, the reversal potentials for the sodium and potassium currents (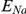 and 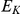, respectively) were also considered unknown. Estimation was based on a simulated recording of membrane potential with duration 2 s. The noise parameters were 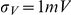 and 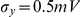, 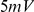, 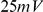 or 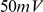. (**A**) Inference of 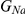, 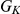, 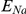 and 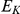 during smoothing. The accuracy of the estimates decreases and their variance increases with increasing observation noise. (**B**) The box plot of the time series in A for 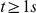. Data were first normalized according to Eq. 35. The reduction in the accuracy and precision at higher levels of observation noise were more prominent in the case of the maximal conductances (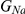 and 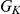) and less prominent in the case of reversal potentials (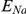 and, particularly 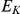). The membrane potential, activation and inactivation variables, intrinsic and observation noise were also subject to estimation, as in Fig. 1. In these simulations, 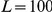, 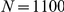, 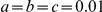 and the prior interval of 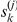 was 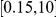.

The results from this set of simulations are summarized in Fig. 5. For 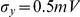, the expectations of the four parameters 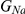, 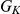, 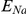 and 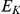 (red solid lines in Figs. 5Ai–iv) eventually converged to their true values (dashed lines in the aforementioned figures). For 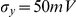, the expectations of these parameters (light red solid lines in Figs. 5Ai–iv) also converged, although the expectations for 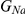 (Fig. 5Ai) and, to a lesser degree, 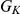 (Fig. 5Aii) deviated noticeably from their true values. As expected, at higher levels of noise, the variance of the final estimates was larger, although the rate of convergence did not seem to be affected, due to the large number of particles we used (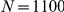; see ceiling effect in Fig. 3Bii). The inferred parameters 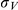 and 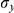 (not illustrated for clarity) followed a similar convergence pattern.

In Fig. 5B, we show, for each tested value of 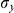, the box plots of the above four parameters, which were computed from the data points (as in Fig. 5A) corresponding to time 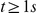. For each parameter and each value of 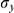, the data were first normalized as follows:
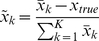
(35)where 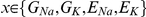. The box plots in Fig. 5B were constructed from the normalized data points 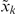. The above normalization was necessary since it made possible the comparison between different data sets, each characterized by its own mean, variance and unit of measurement. In the box plots in Fig. 5B, zero (i.e. the dashed lines) corresponds to the true parameter values, while discrepancies from the true parameter values along the y-axis are given in relation to the average 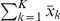. We may observe that for very low levels of observation noise (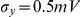), the posteriors of the four examined parameters were clustered tightly around their true values, but for larger levels of noise (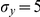, 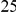 and 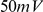), we observed larger discrepancies from the true parameter values and broader inferred posteriors. The parameters following more noticeably this trend were the conductances 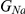 and 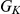, while 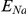 and, particularly, 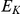 were less affected. This indicates that smoothing is more sensitive to changes in some model parameters than others and this is why these parameters were tightly controlled. In summary, increasing the levels of measurement noise (i.e. the value of parameter 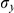) decreased the accuracy and precision of the algorithm, but it did not significantly affect the rate of convergence due to the large number of particles used during the simulations.

### High-Dimensional Inference Problems are Resolved Given Sufficiently Informative Priors

**Figure 6:**
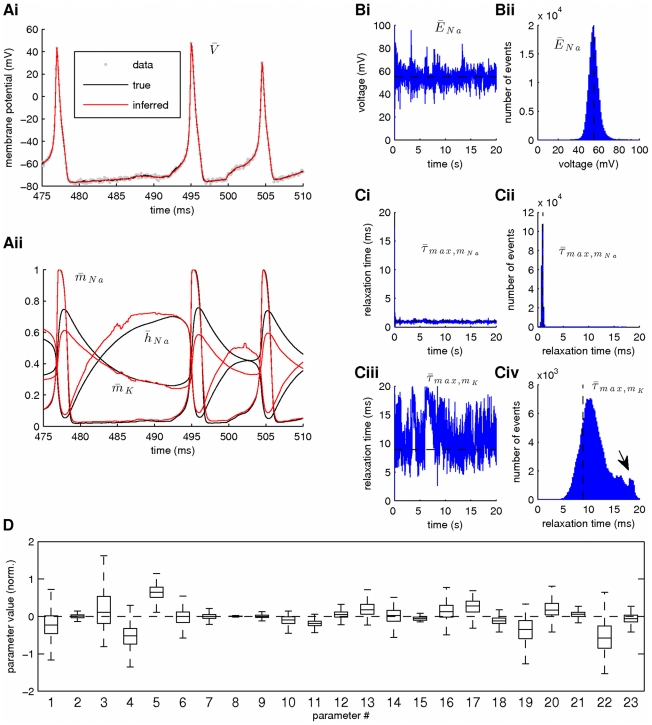
Estimation of all parameters in a single-compartment conductance-based model using the fixed-lag smoother. Estimation was based on a simulated recording of the membrane potential with duration 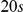. Noise parameters were 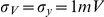. For clarity, only 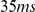 of activity are illustrated in Ai,ii. (**A**) Smoothing of the membrane potential (Ai) and the unobserved activation and inactivation variables for the sodium and potassium currents (Aii). (**B, C**) Estimated posteriors for 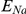 (B), 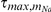 (Ci,ii) and 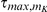 (Ciii,iv). The histograms on the right were constructed form the data on left. (**D**) Box plot of the 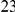 estimated parameter posteriors in the model. These included the standard deviations of intrinsic and observation noise, maximal conductances, reversal potentials and kinetics of all currents in the model (see Table 1). The estimates were first normalized according to Eq. 35. Parameter identification numbers are as in Table 1. In these simulations, 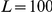, 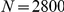, 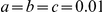 and the prior interval for 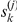 was 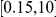.

At the next stage, we treated all parameters in the model (a total of 23 parameters; see Table 1) as unknown. Therefore, the extended state vector took the following (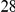-dimensional) form:
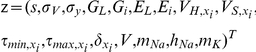
where 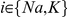 and 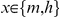. These parameters included the standard deviations of intrinsic and observation noise (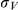 and 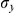, respectively), the maximal conductances 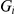 and reversal potentials 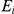 of all currents in the model and the parameters controlling the steady-states and relaxation times of activation and inactivation for the sodium and potassium currents (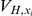, 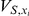, 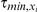, 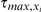 and 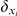). The results from this simulation are illustrated in Fig. 6.

We observed that the true signal (membrane potential) was inferred with very high fidelity (Fig. 6Ai). The sodium activation 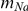 was also recovered with very high accuracy, while estimation of the hidden states 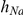 and 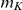 (sodium inactivation and potassium activation, respectively) was also satisfactory (despite significant deviations, the general form of the true hidden states was recovered without any observable impact on the dynamics of the membrane potential), as shown in Fig. 6Aii. Among the 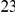 estimated parameters, we illustrate (in Figs. 6B and 6C) the estimated posteriors for the reversal potential of sodium 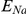 (Fig. 6B) and for parameters 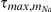 (Figs. 6Ci,ii) and 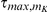 (Figs. 6Ciii,iv), which control the activation of sodium and potassium currents, respectively. We focus on these parameters, because they represent three different characteristic cases. The posteriors of parameters 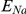 and 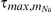 are unimodal (see Figs. 6Bii and 6Cii) and they were estimated with relatively high accuracy. Particularly, the posterior for 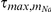 was estimated with very high precision and accuracy, despite its broad prior interval (the y-axis in Fig. 6Ci and the x-axis in Fig. 6Cii). On the other hand, the estimated posterior of 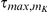 covered a large part of its prior interval (the y-axis in Fig. 6Ciii and the x-axis in Fig. 6Civ), its main mode was located at a slightly larger value than the true parameter value, while at least two minor modes seem to be present near the upper bound of the prior interval (the arrow in Fig. 6Civ). These results reiterate our previous conclusion that smoothing may be particularly sensitive to some parameters, but not to others. The posteriors of parameters in the former category are very precise and narrow (as in the case of 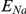 and, especially, 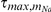), while the parameters in the latter category are characterized by broader posteriors. Also, we can observe that the fixed-lag smoother has the capability to provide a global approximation of the unknown posteriors, including their variance and the location of major and minor modes (i.e. global and local optima). An overview of all inferred posteriors is given by the box plot in Fig. 6D, which was constructed after all data (as in Figs. 6Bi, 6Ci and 6Ciii) were normalized according to Eq. 35. Again, it may be observed that while some of the estimated parameter posteriors are quite precise and accurate, such as 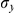 (parameter 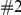), 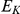 (parameter 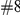) and 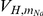 (parameter 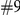), others are less precise and accurate, such as the maximal conductances (parameters 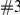 to 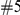), 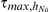 (parameter 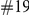) and 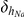 (parameter 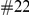).

**Figure 7:**
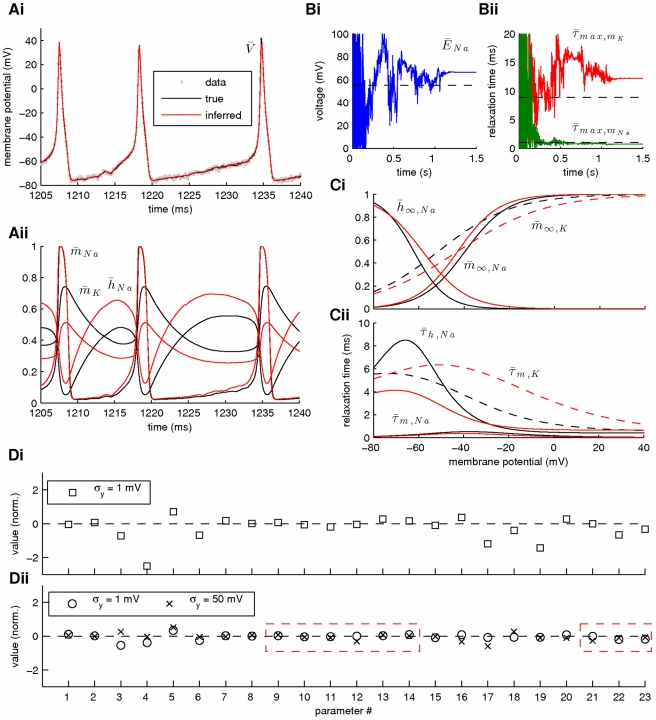
The effect of prior parameter intervals on the accuracy of the fixed-lag smoother. Estimation was based on a simulated recording of the membrane potential with duration 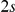. Noise parameters were 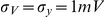. For clarity, only 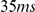 of activity are illustrated in Ai,ii. Unlike Fig. 6, the prior interval for the scaling factors 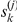 was now assumed equal to 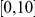. (**A**) Smoothing of the membrane potential (Ai) and the unobserved activation and inactivation variables for the sodium and potassium currents (Aii). (**B**) Estimates for parameters 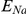 (Bi), 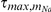 and 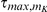 (Bii). Convergence to an optimal parameter vector was achieved after approximately 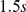 of activity. Notice that this optimal parameter vector falls within the support of the corresponding parameter posteriors (see Figs. 6Bii, 6Cii and 6Civ). (**C**) Inferred steady states (Ci) and relaxation times (Cii) for the activation and inactivation variables of sodium and potassium currents (red lines) against their true counterparts (black lines). (**D**) Inferred parameter values when broad (Di) or narrow (Dii) prior intervals were used for the parameters controlling the kinetics of sodium and potassium ionic currents (see Table 1). Plots A, B and C correspond to plot Di. In Dii, we also illustrate the estimated parameter values when very noisy data were used (see also Supplementary Fig. S3). In these simulations, 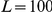, 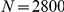 and 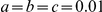.

The simulation results presented above were obtained by assuming a prior interval for the scaling factors 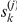 equal to 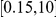. When we repeated the simulation using the prior interval 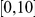, the true underlying membrane potential was again inferred with very high fidelity (Fig. 7Ai), while the hidden states 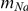, 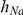 and 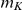 were also estimated with sufficient accuracy (Fig. 7Aii). In this case, however, the estimates of the “unknown” parameters converged to single points in parameter space (as illustrated, for example, for parameters 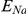, 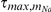 and 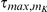 in Figs. 7Bi–ii), which fall within the support of the posteriors illustrated in Figs. 6B and 6C. The activation and inactivation steady states (Fig. 7Ci, red solid lines) and relaxation times (Fig. 7Cii, red solid lines) as functions of voltage, which were computed from these estimates, were also similar to their corresponding true functions, with the curves for 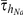 and 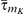 manifesting the largest deviation from truth (black solid lines in Figs. 7Ci,ii). An overview of the estimated parameter values (after normalizing using Eq. 35) is given in Fig. 7Di. As stated previously, some estimates were close to their true counterparts, while others were not. For example, the activation of the sodium current 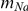 (Fig. 7Aii) and its steady state 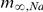 (Fig. 7Ci), which are important for the correct onset of the action potentials, were inferred with relatively high accuracy. On the other hand, larger errors were observed, for example, in the inference of sodium inactivation (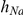; Fig. 7Aii) or in the estimation of 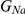 (parameter 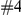; Fig. 7Di), the maximal conductance for the sodium current.

Given the fact that the data on which inference was based (a single noisy recording of the membrane potential) was of much lower dimensionality than the extended state we aimed to infer, the observed discrepancies between inferred and true model quantities were unlikely to vanish unless we imposed more strict constraints on the model. When we repeated the previous simulation using more narrow prior intervals for some of the parameters controlling the kinetics of the sodium and potassium currents in the model (see red dashed boxes in Fig. 7Dii and bold intervals in Table 1), the estimated parameters settled closer to their true values (Fig. 7Dii). This was true even for parameters on which more narrow intervals were not directly applied, such as the maximal conductances (i.e. parameters 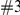 to 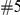 in Fig. 7Dii), and even when data with higher levels of observation noise were used (Fig. 7Dii, data points indicated with crosses; see also Fig. S3). It is important to mention that using more narrow prior constraints only affected the accuracy of the final estimates, not the quality of fitting the experimental data, which in all cases was of very high fidelity. Alternatively, we could have constrained the model by increasing the dimensionality of the observed signal, e.g. by using simultaneously more that one unique voltage traces (each generated under different conditions of injected current) during smoothing. We examine the use of multiple data sets simultaneously as input to the fixed-lag smoother later in the Results section.

In summary, the smoothing algorithm can be used to resolve high-dimensional inference problems. In combination with sufficient prior information (in the form of bounded regions within which parameters are allowed to fluctuate; see Table 1), the fixed-lag smoother can provide estimates of the intrinsic and observation noise, maximal conductances, reversal potentials and kinetics of ionic currents in a single-compartment Hodgkin-Huxley-type neuron model, based on low-dimensional noisy experimental data.

### Parameter Estimation in Compartmental Models is Straightforward Using the Fixed-Lag Smoother

Next, we tested whether the fixed-lag smoother could be successfully applied on inference problems involving more complex models than the one we used in the previous sections. For this reason, we focused on a two-compartment model of a vertebrate motoneuron containing sodium, potassium and calcium currents and intracellular calcium dynamics, which were differentially distributed among a soma and a dendritic compartment [37]. The model (modified appropriately to include intrinsic noise terms) is summarized below:
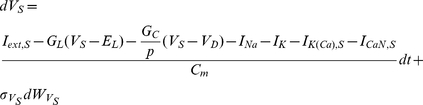
(36)

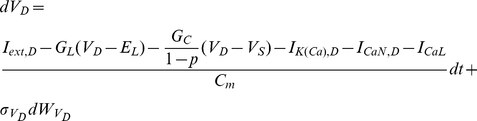
(37)where 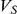 and 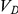 is the membrane potential at the soma and dendritic compartments, respectively, and 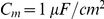. The leakage conductance and reversal potential were 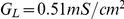 and 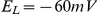, respectively. The coupling conductance was 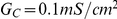 and the ratio of the soma area to the total surface area of the cell was 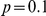. The various ionic currents in the above model were as follows: (a) a transient sodium current, 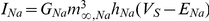, (b) a delayed rectifier potassium current, 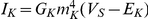, (c) a calcium-activated potassium current, 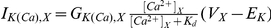, where 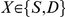 and 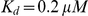 (the half-saturation constant), (d) an N-type calcium current, 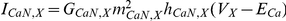, where 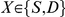 and (e) an L-type calcium current, 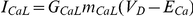. The various activation and inactivation dynamic variables in the above model were modeled using first-order relaxation kinetics (as in Eq. 32), where the various steady states were assumed to be sigmoid functions of voltage (Eq. 33). Notice, that the activation of 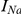 was assumed instantaneous and therefore, it was given at any time by the voltage-dependent steady state 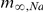. The relaxation times for sodium inactivation and potassium activation were also functions of voltage as in Eq. 34:
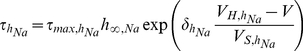
(38)

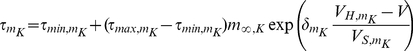
(39)where the parameters 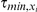, 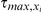 and 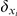 (with 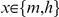 and 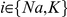) were chosen by fitting the above expressions to the original model in [37]. The relaxation times for the remaining activation and inactivation variables were constant. All parameters values in the model are given in Table 2.

The intracellular calcium concentration at either the soma or the dendritic compartment was also modeled by a first-order differential equation, as follows:
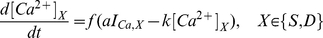
(40)where 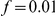, 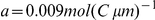 and 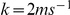. The total calcium current is 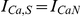 at the soma (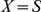) and 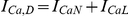 at the dendritic compartment (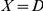).

The observation model assumed simultaneous noisy recordings of the membrane potential from both the soma and dendritic compartments, as follows:
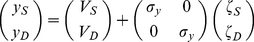
(41)where 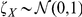 with 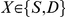. Notice that 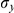 is the same for both compartments.

In the above model, the externally injected currents 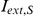 and 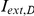 were sequences of random current steps with duration up to 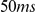 (instead of 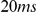 as in the single-compartment model, due to the presence of slower currents in the two-compartment model) and magnitude between 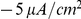 and 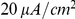. Current was injected in both the dendritic compartment and the soma (instead of just in the soma), because preliminary simulations indicated that this experimental setting facilitated parameter estimation, presumably due to the generation of a more variable (and, thus, information-rich) data set. The injected currents and the induced noisy voltage traces 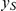 and 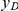 comprised the simulated data on which parameter estimation was based.

**Figure 8:**
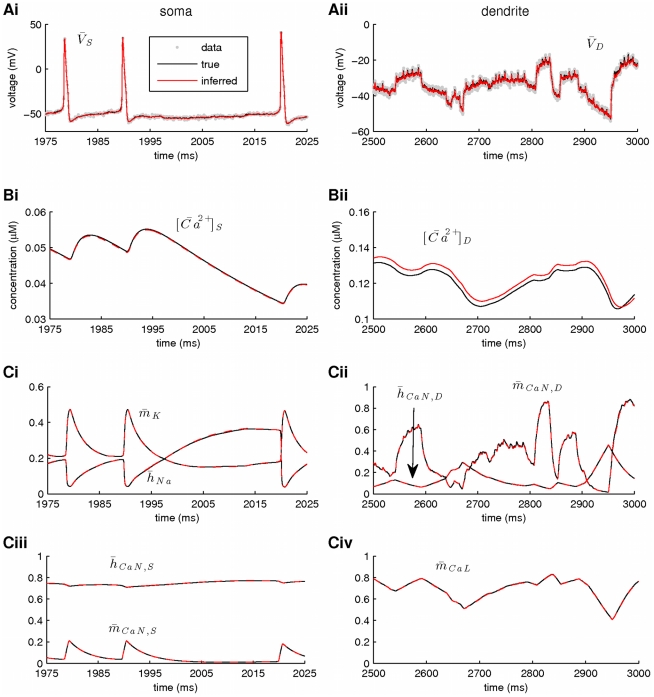
Simultaneous estimation of hidden model states (including intracellular calcium concentrations) and maximal conductances in a two-compartment model of a vertebrate motoneuron (I). Estimation was based on two 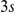-long simulated recordings of the membrane potential, each recorded simultaneously from the soma and the dendritic compartment. Only part of the recorded activity is illustrated in A, B and C for clarity. Notice the different time scales between the right and left panels. (**A**) High-fidelity smoothing of the membrane potential at the soma (Ai) and the dendritic compartment (Aii). (**B**) Inference of the unobserved calcium concentrations at the soma (Bi) and the dendrite (Bii). (**C**) Inference of the unobserved activation and inactivation variables for the sodium and potassium currents (Ci) and the N-type calcium current (Ciii) at the soma and the N-type (Cii) and L-type (Civ) calcium currents at the dendritic compartment. Notice the almost complete overlap between true (black lines) and inferred (red lines) dynamic variables in Ci–iv. This was not surprising since we assumed, at this stage, that the kinetics of all gated currents were known. In these simulations, 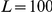, 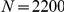, 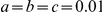 and the prior interval for 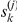 was 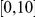.

**Figure 9:**
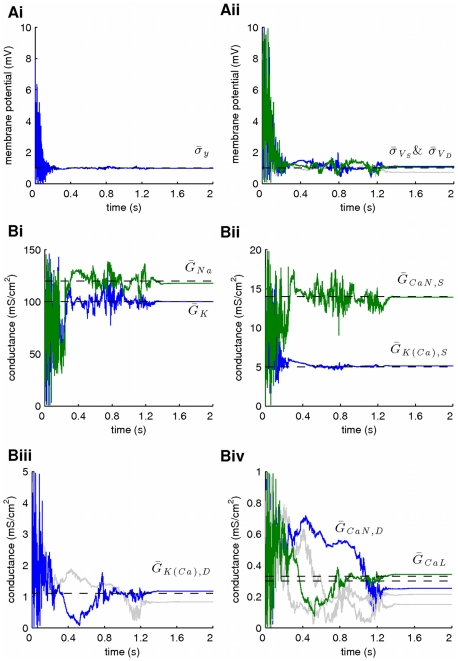
Simultaneous estimation of hidden model states (including intracellular calcium concentrations) and maximal conductances in a two-compartment model of a vertebrate motoneuron (II). Inference of maximal conductances and noise parameters during fixed-lag smoothing. (**A**) The standard deviations of the observation (Ai) and the intrinsic (Aii) noise at the soma and the dendrite. (**B**) Inferred maximal conductances of the sodium and potassium currents at the soma (Bi), of the N-type calcium current and the calcium-activated potassium current at the soma (Bii), of the calcium-activated potassium current at the dendrite (Biii) and of the N-type and L-type calcium currents at the dendrite (Biv). In all cases, parameter expectations gradually converged towards the true parameter values (dashed lines) after less than 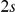. The grey lines in Aii, Biii and Biv correspond to estimated parameters, when current was injected in the soma only. In these simulations, 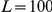, 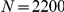, 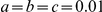 and the prior interval for 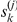 was 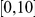.

First, we aimed to infer the noise parameters and maximal conductances of all voltage- and calcium-gated currents in the model, assuming that the kinetics of these currents were known. This implied an extended-state vector with 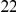 components as shown below
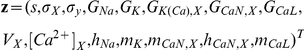
where 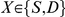. The results from this simulation are illustrated in Figs. 8 and 9. The fixed-lag smoother managed to recover the hidden dynamic states (including the time-evolution of the intracellular calcium; Fig. 8), the standard deviations of the intrinsic and observation noise (Figs. 9Ai,ii) and the true values of all the gated maximal conductances (Figs. 9Bi–iv) in the model using approximately 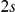 of simulated data and 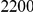 particles. Notice that, in Figs. 8Ci–iv, the inferred hidden gating states (dashed red lines) coincide extremely well with the true ones (solid black lines), which is not surprising, since the voltage-dependent kinetics of these states were assumed known and the true membrane potential at the soma and dendritic compartment was recovered with very high fidelity (Figs. 8Ai,ii). Also, notice that, in Figs. 9Aii, 9Biii and 9Biv, the estimation of the standard deviation of the intrinsic noise, 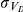, and the maximal conductances of calcium and calcium-dependent currents in the dendritic compartment (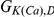, 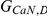 and 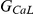) was improved after injecting current in both the soma and the dendritic compartment (compare the grey solid lines, which correspond to injection in the soma only, to the color ones in the aforementioned figures).

**Figure 10:**
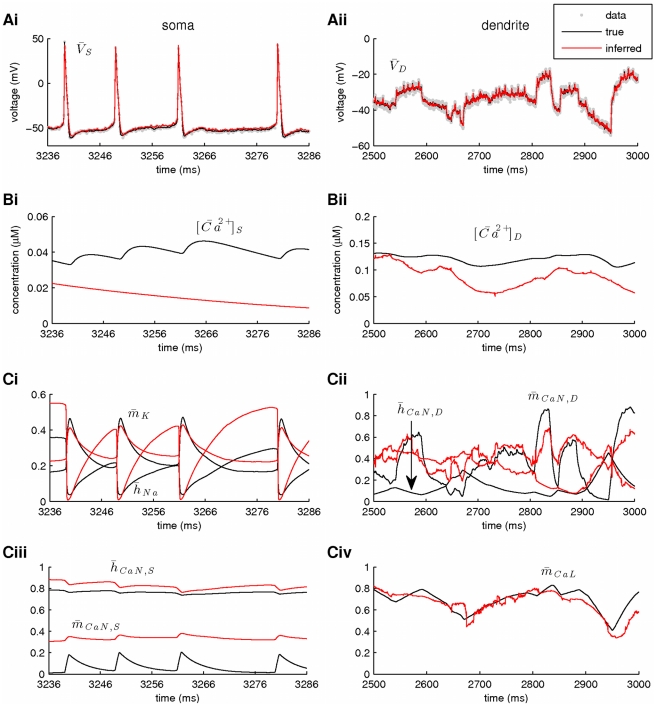
Simultaneous estimation of hidden model states, maximal conductances and kinetic parameters in a two-compartment model of a vertebrate motoneuron (I). Estimation was based on two simulated 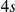-long simultaneous recordings of the membrane potential from the soma and dendritic compartment. Only part of this data is illustrated for clarity. Notice the different time scales between the left and right panels. (**A**) High-fidelity smoothing of the observed voltage at the soma (Ai) and the dendrite (Aii). (**B**) Inference of unobserved calcium concentrations at the soma (Bi) and dendritic compartment (Bii). (**C**) Inference of the unobserved activation and inactivation variables for all voltage-gated currents at the soma and the dendrite. Since the kinetics of voltage-gated currents were assumed unknown, the difference between true (black lines) and inferred (red lines) dynamic variables was significant (compare to Fig. 8). The inferred parameters are shown in Fig. 7Ai. In these simulations, 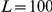, 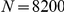, 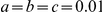 and the prior interval for 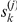 was 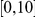.

**Figure 11:**
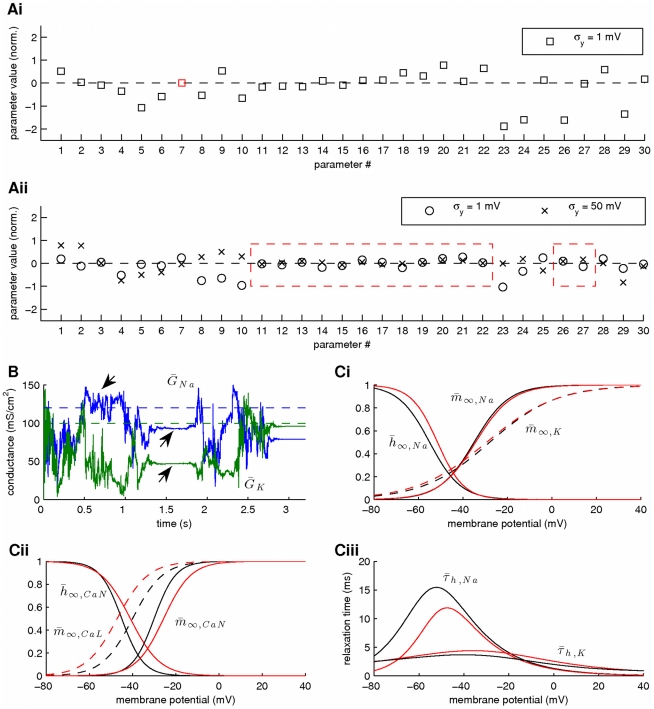
Simultaneous estimation of hidden model states, maximal conductances and kinetic parameters in a two-compartment model of a vertebrate motoneuron (II). Inference of maximal conductances, noise and kinetic parameters during smoothing. (**A**) Inferred parameters in the model using broad or narrow prior intervals and high or low levels of observation noise. Estimates were normalized according to Eq. 35. Parameter identification numbers are as in Table 2. The estimates in Ai were obtained using broad prior intervals (see Table 2). The maximal conductance 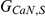 (parameter #7) converged to zero and, for this reason, it is indicated with a red square. These estimates correspond to the results shown in Fig. 10. Estimates in Aii were obtained using narrow prior intervals for some of the parameters controlling the kinetics of ionic currents (see red dashed boxes) at either low (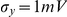) or high (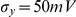) levels of observation noise (see also Supplementary Figs. S4 and S5). (**B**) Inferred maximal conductances for sodium (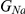) and potassium (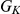) when narrow prior intervals and low levels of observation noise were used (circles in Aii). Notice the temporary convergence of the estimates (arrows) before jumping away towards their final values. (**C**) True (black lines) and inferred (red lines) activation and inactivation steady-states for the sodium and potassium currents (Ci) and the N-type and L-type calcium currents (Cii) and for the relaxation times for sodium inactivation and potassium activation (Ciii), when narrow prior intervals and low levels of observation noise were used (circles in Aii). In these simulations, 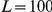, 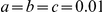 and the prior interval for 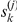 was 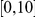. The number of particles was 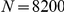 in Ai and 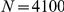 in Aii, B and C (see main text for further comments).

In a second stage, we assumed that the kinetics of all voltage-gated ionic currents were also unknown, implying an extended state vector with 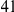 components, as follows:
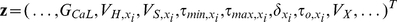
where 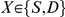, 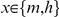 and 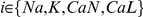. Our results from this simulation are summarized in Figs. 10 and 11. Again, the membrane potential at the soma and the dendrite were inferred with very high fidelity (Fig. 10Ai,ii). However, the estimated hidden dynamics of most ionic currents and intracellular calcium concentrations in the model deviated significantly from their true counterparts (Fig. 10B,C). The expectations of all estimated parameters are illustrated in Fig. 11Ai. As in the case of the single-compartment model, by imposing tighter prior constraints on some of the parameters controlling the kinetics of ionic currents in the model (see red dashed box in Fig. 11Aii and Table 2), we managed to reduce the discrepancies of the estimates from their true values (Fig. 11Aii and Supplementary Fig. S4). This was true even for parameters on which stricter priors were not directly applied. The inference was completed after processing almost 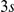 of data, as shown in Fig. 11B for the maximal conductances of sodium and potassium currents at the soma. Interestingly, the algorithm seems to temporarily settle at local optima (see arrows in Fig. 11B) before “jumping” away and, eventually, converge at the final estimates. The inferred voltage-dependent steady-states of the sodium, potassium and calcium currents (Figs. 11Ci,ii) and the relaxation times for the sodium inactivation and potassium activation (Fig. 11Ciii) were also very similar to their true corresponding functions. The algorithm remained operational when more noisy data were used, as illustrated in Fig. 11Aii and in Supplementary Fig. S5.

An interesting fact regarding the simulation results presented in Figs. 10 and 11Ai was that, in order to obtain high-fidelity estimates of the true membrane potential at the soma and dendritic compartment (as shown in Figs. 10Ai,ii) we had to use more than 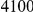 particles, the number calculated by the 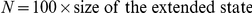 rule (see Methods). In particular, we used 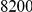 particles, although we cannot exclude that a smaller number may have sufficed. After applying more narrow prior constraints (Figs. 11Aii, B, C, S4 and S5), using the number of particles calculated by the above simple heuristic (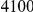 in this case) was again sufficient for accurately inferring the true membrane potential (see Fig. S4Ai,ii and S5Ai,ii). This implies that as the complexity (and dimensionality) of the estimation problem increases, a non-linearly growing number of particles may be required in order to obtain acceptable results, but this situation may be compensated for by providing highly informative priors. It should be mentioned that the two-compartment model allows for the physical separation of currents and as such it is a slightly better approximation of a real neuron with differential expression of individual currents in different cellular compartments. However, in no way does it capture the full morphological complexity of a real neuron. As such, current injection into the dendritic compartment can not be replicated accurately in a real neuron, as current injection in the model will have a uniform effect on all currents in that compartment, whilst current injection into the dendrite of a neuron would have far more complex effects on dendritic currents, which potentially would be dependent on the distance from the injection site. Thus, whilst it would be possible, albeit challenging, to carry out dual recordings from the soma and dendrites in a real neuron this would not be the same as the dual current injection in the model. In this case, application of the fixed-lag smoother on a more spatially detailed model would be necessary (and feasible). In principle, the method can also assimilate other types of spatial data, such as calcium imaging data, in case recordings from multiple neuron locations are not available (although we do not examine this case in detail in this paper).

Given the large number of unknown parameters and hidden states in combination with the low dimensionality of the data (notice that the intracellular calcium concentration was assumed unobserved), it was truly remarkable that the algorithm managed to recover much of the extended state vector with relatively satisfactory accuracy. However, it should be noted that in our simulations we assumed knowledge of important information, such as the passive conductances 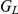 and 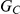 and the reversal potentials of sodium, potassium and calcium currents. This and the fact that the availability of prior information in the form of more narrow parameter boundaries improved significantly the accuracy of the final estimates emphasizes our previous conclusion that prior information is important for the successful inference of unknown model parameters and hidden model states using the fixed-lag smoother. Given such information, inference in complex compartmental models based on simultaneous recordings from several neuron locations and, possibly, measurements of intracellular calcium, can be naturally achieved via appropriate formulation of the extended state vector and application of the fixed-lag smoother.

### Parameters in a Model of an Invertebrate Motoneuron were Inferred from Actual Electrophysiological Data Using the Fixed-Lag Smoother

In a final set of simulations, we applied the smoother on actual electrophysiological data in order to estimate the unknown parameters in a single-compartment model of the B4 motoneuron from the nervous system of the pond snail, *Lymnaea stagnalis* [38]. This neuron is part of a population of motoneurons, which receive rhythmic electrical input from upstream Central Pattern Generator interneurons and in turn innervate and control the movements of the feeding muscles via which the animal captures and ingests its food. Previous studies in these neurons have demonstrated the presence of a transient inward sodium current 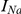, a delayed outward potassium current 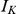 and a transient outward potassium current 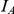 [41]. A hyperpolarization-activated current 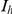 was conditional on the presence of serotonin in the solution [38] and, therefore, this current was not included in this instance of the B4 model. Thus, the current conservation equation for a single-compartment model of the B4 motoneuron (appropriately modified to include an intrinsic noise term) took the following form:
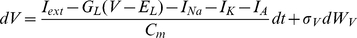
(42)where the leakage conductance, leakage reversal potential and membrane capacitance in the above model were estimated *a priori* based on neuron responses to negative current pulses (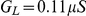, 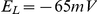 and 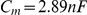, respectively). The voltage-activated currents that appear in the above expression were modeled as follows: (a) 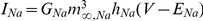, (b) 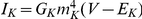 and (c) 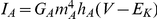, where 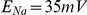 and 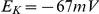 as in [41]. The dynamic activation and inactivation variables of these currents (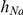, 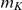, 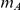 and 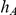) obeyed first-order relaxation kinetics (as in Eq. 32) with voltage-dependent steady-states (Eq. 33) and relaxation times (Eq. 34 with 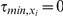 and 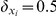), similarly to previously published neuron models in the central nervous system of *Lymnaea* [42]. The observation model was as in Eq. 7.

**Figure 12:**
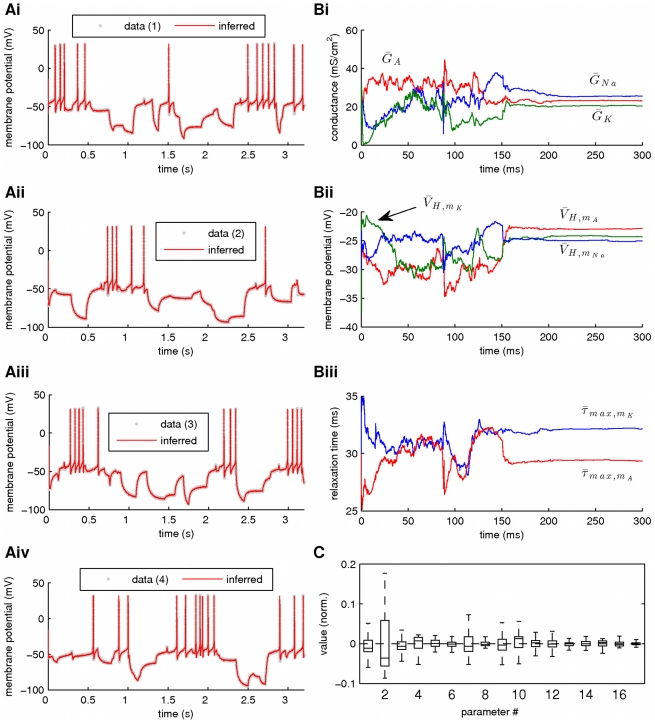
Parameter estimation in a model invertebrate motoneuron based on actual electrophysiological data. Estimation was based on four independent 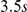-long recordings of the membrane potential from the same B4 motoneuron. (**A**) Simultaneous, high-fidelity smoothing of the four membrane potential recordings. (**B**) A total of 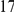 free parameters in the model were inferred during smoothing (see Table 3), including the maximal conductances of the transient sodium and potassium and persistent potassium currents (Bi), the half steady-state activation values (Bii) and the relaxation times for the activation of the potassium currents (Biii). The remaining inferred parameters are not illustrated for clarity, but they follow a similar convergence pattern. (**C**) Box plot of all inferred parameters in the model. Parameter identification numbers are as in Table 3. Estimates were normalized as explained in the main text (the non-normalized mean parameter values are given in Table 3). In this simulation, 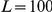, 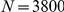, 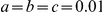 and the prior interval for 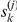 was 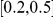.

The raw data we used for inferring the parameters in the above model took the form of four independent 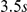-long recordings of the membrane potential from the same B4 motoneuron. Each recording was taken while injecting an external current in the neuron consisting of a sequence of random steps ranging in amplitude between 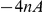 and 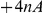 and with duration between 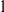 and 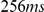. A particular characteristic of the data generated under these conditions was the presence of brief bursts of spikes, which were interrupted by relatively long intervals of non-activity (corresponding to sub-threshold excitatory and inhibitory current injections, respectively; see Figs. 12Ai–iv). These long intervals of inactivity were not informative and they negatively affected the performance of the smoother by permitting the random drift of particles towards non-optimal regions of the parameter space (see Supplementary Fig. S6). However, when the four recordings are considered together, the intervals of inactivity at any single voltage trace overlap with intervals of activity at the remaining three voltage traces, resulting in a four-dimensional data set, where the overall intervals of inactivity were minimized. This four-dimensional data set was used as input to the smoother during the inference phase.

Thus, the 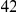-dimensional extended state vector became:
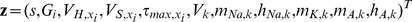
where 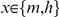, 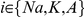 and 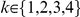. Notice the presence of four groups of hidden dynamic states, {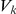, 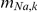, 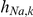, 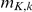, 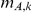, 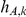}, where each group corresponds to a different voltage trace (and associated externally injected current, 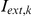). The evolution of all four groups of dynamic variables was governed by a common (shared) set of parameters. In total, we had to estimate 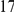 unknown parameters. The boundaries within which the values of these parameters were allowed to fluctuate are given in Table 3 (indicated in bold) and they were chosen from within the support of the posteriors in Supplementary Fig. S7 (after a few trial-and-error simulations), which were obtained by using the broader prior intervals given in Table 3. Notice that the marginal distributions illustrated in Fig. S7 have large variance and multiple modes and, although they provide a global view of the structure of the parameter space, they cannot be used to identify a single combination of optimal parameters values, since they do not include any information regarding correlations between parameters. Using the major modes of the inferred posteriors did not lead to an accurate (or even spiking) predictive model. Thus, the estimation was based on using more narrow prior intervals, which helped us estimate unimodal posteriors with small variance (see Fig. 12C) and, thus, identify a single combination of optimal parameters that could be used in predictive simulations. We cannot prove that other optimal combinations of parameters do not exist, but we were not able to find any (i.e. by choosing different narrow prior intervals) after a reasonable amount of time. Also, notice that the standard deviations of the intrinsic and observation noise were not subject to estimation, but instead they were given (through trial and error) the minimal fixed values 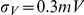 and 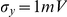, respectively. If left free during smoothing, the values of these parameters fluctuated uncontrollably, masking the contribution of the remaining parameters in the model and, thus, achieving an almost perfect (but meaningless) smoothing of the experimental data. This is an indication that the B4 model we used may be missing one or more relevant components, such as additional currents and compartments (see below for further analysis of this point). We did not observe this effect in the cases examined in the previous sections, where simulated data was used, because the models responsible for the generation of this data were, by definition, precisely known.

Our results from this set of simulations are illustrated in Fig. 12. Simultaneous smoothing of all four data sets was again accomplished with high fidelity, as illustrated in Figs. 12Ai–iv. The artificial evolution of the expectations of the conductances for the transient sodium, persistent potassium and transient potassium currents, as well as of some of the kinetic parameters that were estimated in the model is illustrated in Figs. 12Bi–iii. The distributions of all inferred parameters (normalized after replacing 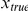 in Eq. 35 with 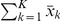, for each tested parameter) are also illustrated in Fig. 12C. The inferred expectations of all parameters are given in Table 3.

**Figure 13:**
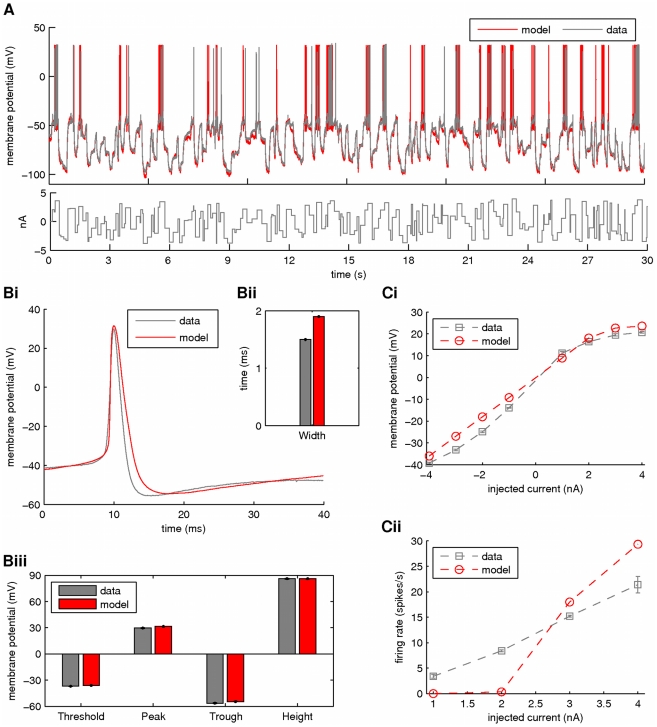
Comparison between B4 model activity and the biological neuron. (**A**) Response of the model and the biological B4 motoneuron to a sequence of current steps with random amplitude and duration. Current step amplitudes were from 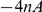 to 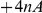 and current step durations from 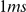 to 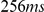. Intrinsic and observation noise in the model were 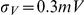 and 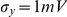, respectively. (**B**) Comparison between model and biological B4 action potentials. The width of the spikes was measured at half their peak amplitude. (**C**) Current-Voltage (IV) and Current-Frequency (IF) relations for the model and biological B4 neurons. In order to construct these relations both the model and biological neurons were injected with 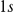-long current pulses with amplitude between 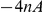 and 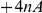.

In order to examine the predictive value of the model given the estimated parameter expectations in Table 3, we compared its activity to that of the biological B4 neuron, when both were injected with a 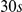-long random current consisting of a sequence of current pulses with amplitude ranging from 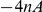 to 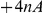 and duration from 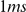 to 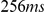. Our results from this simulation are illustrated in Fig. 13. We observed that the overall pattern of activity of the model was similar to that of the biological neuron (Fig. 13A). Whilst the model overall generated more action potentials, some individual spikes were absent in the simulated data. A more detailed examination of our data revealed specific differences between the biological and model neurons, which explain the differences in the overall activity between the two (Fig. 13B, C). The spike shape of the model neuron was quite similar to that of its biological counterpart (Fig. 13Bi), including spike threshold, peak, trough and height (i.e. trough-to-peak amplitude; Fig. 13Biii), but the simulated spike had a slightly longer duration than the biological one (half-width: 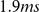 vs 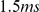; Fig. 13Bii).

In a second set of experiments, both the biological and model neurons were injected with 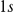-long current pulses ranging from 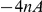 to 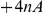 and their current-voltage (IV) and current-frequency (IF) relations were constructed (Fig. 13C). The IV plot showed some non-linear behavior in response to negative current pulses in the experimental data (probably due to the presence of a residual 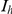 current), which was not present in the simulations (Fig. 13Ci). As a result, the slope of the part of the IV curve corresponding to 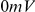 was more shallow in the simulations than in the experimental data. Moreover, the rheobase was lower in the experimental data than in the model, but the slope of the IF curve was steeper in the simulated data, which resulted in higher firing rates for the model at injected currents larger than approximately 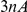 (Fig. 13Cii). This feature can account for the overall level of spiking in the model neuron when compared to the biological one (Fig. 13A).

Overall, this analysis illustrates that the assumed B4 model did not capture all the aspects of the real neuron. However, this does not mean that our estimation method is flawed. It just shows that the model is actually missing some relevant components, such as additional ionic currents or compartments, which would be necessary for approximating more accurately the spatial structure and biophysical properties of the biological neuron. In the first part of the manuscript we have demonstrated that if the underlying model is complete, then our method produced accurate estimates of the true parameter values, given sufficient informative priors. Thus, it is safe to assume that the observed differences between the biological and model neurons can be minimized, if the fixed-lag smoother is applied on a more complex model of the B4 motoneuron.

In summary, we used the fixed-lag smoother to estimate the unknown parameters in a single-compartment model of an invertebrate motoneuron based on actual electrophysiological data. The model, although a simplification of the actual biological system, was still quite complex containing a number of non-linearly interacting components and a total of 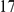 unknown parameters. By using the methodologies outlined in the previous sections, we managed to estimate the values of these parameters, such that the resulting model mimicked with satisfactory accuracy the overall activity of its biological counterpart. Furthermore, we demonstrated the flexibility of the fixed-lag smoother by showing how it can be used to process simultaneously multiple data sets, given an appropriate formulation of the extended state vector.

## Discussion

Parameter estimation in conductance-based neuron models traditionally involves a global optimization algorithm (for example, an evolutionary algorithm), usually in combination with a local search method (such as gradient descent), in order to find combinations of model parameters that minimize a pre-defined cost function. In this paper, we have addressed the problem of parameter estimation in Hodgkin-Huxley-type models of single neurons from a different perspective. By adopting a hidden-dynamical-systems formalism and expressing parameter estimation as an inference problem in these systems, we made possible the application of a range of well-established inference methods from the field of Computational Statistics. Although it is usually assumed that the kinetics of ionic currents in a conductance-based model are known *a priori*, here we assumed that this was not the case and, typically, we estimated kinetic parameters, along with the maximal conductances and reversal potentials of ionic currents in the models we examined.

The particular method we used was Kitagawa's self-organizing state-space model, which was implemented as a fixed-lag smoother. The smoother was combined with an adaptive algorithm for sampling new sets of parameters akin to the Covariance Matrix Adaptation Evolution Strategy. Alternatively, we could have approximated the smoother distribution (Eq. 13) with a two-pass algorithm, consisting of a forward filter followed by a backward smoothing phase, which would make use of the precomputed filter [34]. This would require storing the filter for the whole duration of the smoothed data, which in turn would have very high memory requirements when large numbers of particles or high-dimensional problems are considered. In contrast, the fixed-lag smoother has the advantage that only the particles up to 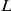 time steps in the past need to be stored, which is less demanding in memory size and computationally more efficient. Moreover, the fixed-lag smoother, being a single-pass algorithm, was more natural to use in the context of on-line parameter estimation.

The applicability of the algorithm was demonstrated on a number of conductance-based models using noisy simulated or actual electrophysiological data. In a recent study, it was found that increasing observation noise led to an increase in the variance of parameter estimates and a decrease in the rate of convergence of the algorithm [28]. Similarly, we observed that at high levels of observation noise, although the algorithm remained functional, its accuracy and precision were reduced (Fig. 5). It is emphasized that, at a particular level of observation noise, the outcome of the algorithm is an approximation of the posterior distributions of hidden states and unknown parameters in the model, given the available experimental data and prior information. In general, these approximate posteriors provide an overview of the structure of the parameter space and they potentially have multiple modes (or local optima). By taking advantage of the adaptive nature of the fixed-lag smoother (and, in particular, by controlling the scaling factor that determines the width of the proposal distribution in Eq. 25), we managed to reduce the variance of these posteriors and, in the limit case, we could force the algorithm to converge to a single optimal point (belonging to the support of the parameter posteriors), which could subsequently be used in predictive simulations (e.g. see Figs. 7D and 11A). Unlike the study in [28], we did not observe any significant reduction in the rate of convergence of the algorithm at high levels of observation noise, which was attributed to a ceiling effect due to the large number of particles we used in our simulations (typically, 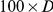, where 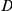 was the dimensionality of the estimation problem; see Figs. 3B and 4C). Thus, we cannot exclude observing such a reduction in the rate of convergence, if a smaller number of particles is used and/or problems of higher dimension are examined. Furthermore, the proposed method requires only a single forward pass of the experimental data, instead of multiple passes, as in the case of off-line estimation methods, including the Expectation Maximization (EM) algorithm. On the other hand, this means that, in general, the proposed algorithm requires processing longer data time series in order to converge. In addition, unlike off-line estimation methods, it does not take into account the complete data trace at each iteration, but at most 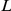 past data points (but, also, see [36] for a partial “remedy” of this situation). In principle, it would be possible to combine previous work on parameter estimation (e.g. [43], [44]) within an EM inference framework in order to estimate various types of parameters (including maximal conductances and channel kinetics) in conductance-based neuron models. This could be an interesting topic for further research.

Our main conclusion was that, using this algorithm and a set of low-dimensional experimental data (typically, one or more traces of membrane potential activity), it was possible to fit complex compartmental models to this data with high fidelity and, simultaneously, estimate the hidden dynamic states and optimal values of a large number of parameters in these models. Based on simulation experiments using simulated data, we found that the estimated optimal parameter values and hidden states were close to their true counterparts, as long as sufficient prior information was made available to the algorithm. This information took the form of knowledge of the values of particular parameters (for example, the passive properties of the membrane) or of relatively narrow ranges of permissible parameter values. Such prior information could have included the kinetics of the ion currents that flow through the membrane or the spatial distribution of various parameter values along different neuron compartments (e.g. the ratio of maximal conductance A between compartment 1 and compartment 2). In real-life situations, such information may become available through current- or voltage-clamp experiments. For example, the passive properties in the B4 model (membrane capacitance, leakage maximal conductance and reversal potential) were inferred from current-clamp data and, thus, they were fixed during the subsequent smoothing phase.

It has been demonstrated that this requirement for prior information may be relaxed, if the data set used as input was sufficiently variable to tease apart the relative contribution of different parameters in a model [15]. A well-established result in conductance-based modeling is that the same pattern of electrical activity may be produced by different parameter configurations of the same model [6]–[9]. This implies that it is impossible to identify, during the course of an optimization procedure, a unique set of parameters using just this single pattern of activity as input to the method. For example, as we observed in the case of the B4 model, the posteriors of the estimated parameters may be characterized by multiple modes (i.e. local optima) or quite large variances, which makes identification of a unique set of optimal parameter values for use in predictive simulations rather difficult (Supplementary Fig. S7). A more variable data set would be necessary in order to constrain the model under study, thus forcing the optimization process to converge towards a unique solution. It should be noted that this conclusion was reached by treating as unknown only the maximal conductances in a conductance-based model [15]. Although it is reasonable to assume that this holds true when the kinetics of ion channels are also treated as unknown, it still needs to be demonstrated whether the generation of a data set sufficiently variable to constrain both the maximal conductances and kinetics of ion channels in a complex conductance-based model is practical or even feasible. A more pragmatic approach would be to rely on a mixture of prior information and one or more sufficiently variable electrophysiological recordings as input to the optimization algorithm. It was shown in this study that both the injection of prior information (in the form mentioned above) and the simultaneous assimilation of multiple data sets is straightforward using the proposed algorithm.

It is important to notice that, unlike more traditional approaches, explicitly defining a cost or fitness function was not required by the fixed-lag smoother. Given the fact that the efficiency of any optimizer can be seriously impeded by a poorly designed cost function, bypassing the need to define such a function may be viewed as an advantage of the proposed method. As in previous studies [43], [44], here lies the implicit assumption that by fitting (or smoothing) with high fidelity the raw experimental data (for example, one or more recordings of the membrane potential), the estimated model would capture a whole range of features embedded in this data, such as the current-frequency response of the neuron. Although this is a reasonable assumption, we found that it did not hold completely true, when our knowledge of the form of the underlying model was not exact, as in the case of the B4 neuron. In this case, although we could achieve a very good smoothing of the experimental data, subsequent predictive simulations using the inferred model parameters revealed discrepancies between simulation output and experimental data. It is likely that these discrepancies will be minimized, if important missing components are added to the model, such as additional ionic currents or, importantly, an approximation of the spatial structure of the biological neuron.

An important outcome of this study was to demonstrate the intimate relation between the self-organizing state-space model and evolutionary algorithms. When used for parameter estimation, the self-organizing state-space model undergoes at each iteration a process of new particle (individual) generation (mutation/recombination) and resampling (selection and multiplication), which parallels similar processes in evolutionary algorithms. At the root of this parallelism is the fact that we need to impose an artificial evolution on model parameters as part of the formulation of the self-organizing state-space model (see Methods), thus providing a unique opportunity to merge the two classes of algorithms. Here, we decided to combine the self-organizing state-space model with an adaptive algorithm similar to the Covariance Matrix Adaptation Evolution Strategy [30] and by following this adaptive strategy, we managed to achieve a dramatic reduction in the variance of parameter estimates. However, this choice is by no means exclusive and other evolutionary algorithms may be chosen instead, e.g. the Differential Evolution algorithm [31]. This is a topic open to further exploration. Notice that, similarly to Evolutionary Algorithms, the proposed method has, in principle, the ability to estimate the possibly multi-modal posterior distribution of the unknown parameters in the examined model, i.e. it is a global estimation method (for example, see Fig. 6C, 11B and S7). At each iteration, the algorithm retains a population of particles, which are characterized by a degree of variability and, thus, give the algorithm the opportunity to randomly explore a wide range of the parameter space, spending on average more time in the vicinity of optimal regions. By imposing narrow prior constraints on some of the unknown parameters, we are effectively reducing the dimensionality of the problem and we force the algorithm to converge towards a particular optimum, which can be later used in predictive simulations.

A point of potential improvement concerns our choice of the proposal density, 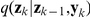. Here, we made the common and straightforward choice to use the transition density 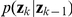 as our proposal. However, the modeler is free to make other choices. For example, a recent study demonstrated that the efficiency of particle filters can be significantly increased by conditioning the proposal density on future observations [36].

An important practical aspect of the proposed algorithm was its high computational cost. This cost increased as a function of the number 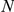 of particles used during smoothing, the length of the fixed smoothing lag 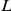, the complexity of the model and the number of unknown parameters in the model. Our simulations on an Intel dual-core i5 processor with four gigabytes of memory took from a few minutes to more than 12 hours to complete. An emerging trend in Scientific Computing is the use of modern massively parallel Graphics Processing Units (GPUs) in order to accelerate general purpose computations, as those presented in this paper. The utility of this approach in achieving significant accelerations of Monte Carlo simulations has been recently demonstrated [45] and it has even been applied recently on parameter estimation problems in conductance-based models of single neurons [46]. Preliminary results using a GPU-accelerated version of the fixed-lag smoother (data not shown) have indeed demonstrated reduced simulation times, but the accelerations we observed were not as dramatic as those reported in the literature [45], [46]. This can always be attributed to the fact that our implementation of the algorithm was not optimized. On the other hand, we observed significant accelerations in our simulations involving the serial implementation of the fixed-lag smoother, just by switching from an open-source compiler (GNU) to a commercial one (Intel), which presumably emitted better optimized machine code for the underlying hardware. Nevertheless, the use of GPUs for general purpose computing is becoming common and it is likely to become quite popular with the advent of cheaper hardware and, importantly, more flexible and programmer-friendly Application Programming Interfaces (APIs).

Overall, our results point towards a generic four-stage heuristic for parameter estimation in conductance-based models of single neurons: (a) First, the general structure of the model is decided, such as the number of ionic currents and compartments it should include. (b) Second, prior information is exploited in order to fix as many parameters as possible in the model and tightly constrain the remaining ones. For example, the capacitance, reversal potentials and leakage conductance in the model may be fixed to values estimated from current-clamp data. By further exploiting current– and voltage-clamp data, narrow constraints may be imposed on the remaining free (e.g. kinetic) parameters in the model. (c) At a third stage, more precise parameter value distributions are estimated by applying the fixed-lag smoother on current-clamp data, such as one or more recordings of the electrical activity of the membrane induced by random current injections. (d) Finally, the predictive value of the model is assessed through comparison to independent data sets and the model is modified, if necessary. It is important to notice that the techniques outlined in this paper are applicable on a wide range of research domains and that they provide a disciplined way to merge complex stochastic dynamic models, noisy data and prior information under a common inference framework.

In conclusion, the class of statistical estimation methods, which the algorithm presented in this paper belongs to, in combination with Monte Carlo approximation techniques are particularly suitable to address high-dimensional inference problems in a disciplined manner. This makes them potentially useful tools at the disposal of biophysical modelers of neurons and neural networks and it is predicted that these methodologies will become more popular in the future among this research community.

## Supporting Information

Figure S1
**Simultaneous estimation of hidden states and channel noise in a stochastic single-compartment model.** Estimation was based on a simulated 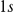-long recording of membrane potential generated by Supplementary Eqs. S1, S2 and S4. For clarity, only 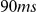 of activity are shown in Figs. Ai,ii. Notice that in these simulations, we assumed the absence of synaptic input (i.e. 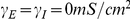). Activity in the model neuron was driven by a random sequence of current steps 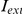 with amplitude between 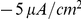 and 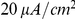 and duration up to 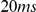. (**A**) Simultaneous inference of the observed membrane potential (Ai) and the hidden activation (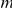, 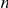) and inactivation (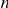) gating variables for the sodium and potassium currents. (**B**) Inference of the standard deviation of the observation noise 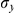 (Bi) and the parameters 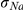 and 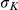, which control the variance of the sodium and potassium channel noise (Bii). Estimates converged to their final values after approximately 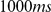. The dashed lines indicate the true values of these parameters. The y-axes in Bi,ii indicate the width of the prior intervals imposed on the corresponding parameters. Simulation parameters were: 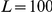, 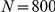 and 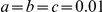. The prior interval for the scaling factors 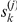 was 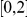.(TIFF)

Figure S2
**Simultaneous estimation of hidden states, channel noise and presynaptic firing rates in a stochastic single-compartment model.** Estimation was based on a simulated 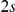-long recording of membrane potential generated by Eqs. S1, S2 and S4 with 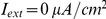. For clarity, only 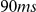 of activity are shown in Figs. Ai,ii. (**A**) Simultaneous inference of the observed membrane potential (Ai) and the hidden activation (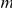, 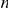) and inactivation (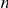) gating variables for the sodium and potassium currents (Aii). (**B**) Inference of the standard deviation of the observation noise 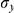 (Bi), parameters 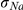 and 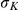, which control the variance of the sodium and potassium channel noise (Bii) and the presynaptic firing rates 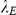 and 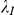 (Biii). Estimates converged to their final values after approximately 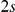 of activity. The dashed lines indicate the true values of the parameters. The y-axes in B and C indicate the width of the prior intervals imposed on the corresponding parameters. Discrepancies from the true values in B are due to the overlapping effects of different parameters controlling observation, channel and synaptic noise. Simulation parameters were: 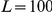, 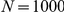 and 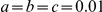. The prior interval for the scaling factors 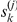 was 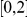.(TIFF)

Figure S3
**Simultaneous inference of hidden states and unknown parameters in the single compartment model (see main text) at high levels of observation noise.** This figure corresponds to Fig. 7Dii in the main text for 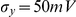. (**A**) Inferred membrane potential (Ai) and unobserved gating variables (Aii). (**B**) Examples of simultaneously inferred parameters, such as maximal conductances (Bi,ii) and reversal potentials (Biii,iv). The y-axes in Bi–iv indicate the prior intervals of the corresponding parameters. Simulation details are as in Fig. 7 in the main text.(TIFF)

Figure S4
**Simultaneous inference of hidden states in the two-compartment model (see main text) at low levels of observation noise.** This figure corresponds to Figs. 11Aii for 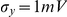, 11B and 11C in the main text. (**A**) Inference of the membrane potential at the soma (Ai) and the dendritic compartment (Aii). (**B**) Inference of the unobserved concentration of intracellular calcium at the soma (Bi) and the dendritic compartment (Bii). (**C**) Inference of the unobserved gating variables for the sodium and potassium currents at the soma (Ci), the N-type calcium current at the soma (Ciii), the N-type calcium current at the dendritic compartment (Cii) and the L-type calcium current at the dendritic compartment (Civ). Simulation details are as in Fig. 11 in the main text.(TIFF)

Figure S5
**Simultaneous inference of hidden states in the two-compartment model (see main text) at high levels of observation noise.** This figure corresponds to Fig. 11Aii for 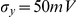. (**A**) Inference of the membrane potential at the soma (Ai) and the dendritic compartment (Aii). (**B**) Inference of the unobserved concentration of intracellular calcium at the soma (Bi) and the dendritic compartment (Bii). (**C**) Inference of the unobserved gating variables for the sodium and potassium currents at the soma (Ci), the N-type calcium current at the soma (Ciii), the N-type calcium current at the dendritic compartment (Cii) and the L-type calcium current at the dendritic compartment (Civ). Simulation details are as in Fig. 11 in the main text.(TIFF)

Figure S6
**Inference in the B4 model using a single recording of the membrane potential.** A single 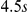-long recording of B4 activity induced by injecting a sequence of random current steps in the neuron was used during smoothing. Random current amplitude was between 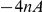 and 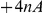 and random step duration was between 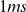 and 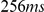. (**A**) Inference of the membrane potential (Ai) and the unobserved gating variables for the sodium and potassium currents in the model (Aii). (**B**) Examples of simultaneously inferred model parameters: maximal conductances of all currents (Bi), half steady-state activation voltages for all currents (Bii) and maximal relaxation times for the activation of the potassium currents in the model (Biii). Notice that in all cases the parameter estimates converge exactly to the middle of their prior intervals (indicated by the y-axes in Bi–iii). This convergence takes place while the algorithm processes the “inactive” region of the data (approximately, from second 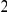 to second 3 in Ai). Based on these converged estimates, the model incorrectly emits spikes later during smoothing (see arrows in Ai), indicating that the estimated parameters are not optimal for smoothing during the whole duration of experimental data. Simulation parameters were as follows: 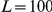, 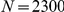 and 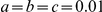. The prior interval for the scaling factors 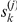 was 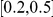. For the 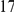 free parameters in the model, we used the narrow prior intervals in Table 3.(TIFF)

Figure S7
**Inferred posterior distributions of all unknown parameters in the B4 model using the broad prior intervals in** **Table 3** **.** Inference was based on simultaneously smoothing four 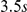-long voltage recordings from the B4 neuron as in Fig. 12A in the main text. As in that case, data smoothing was accomplished with very high fidelity, as illustrated in Fig. 12A. (**A**) Inferred maximal conductances. (**B**) Inferred half steady-state activation and inactivation voltages. (**C**) Inferred activation (Ci) and inactivation (Cii) voltage sensitivities (parameters 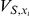 in the model). (**D**) Activation and inactivation relaxation times. The x-axes in all plots indicate the prior parameter intervals we used (Table 3). Notice that most posteriors are very broad (covering a large portion of the prior interval) and not unimodal. Simulation parameters were as described in Fig. 12 of the main text.(TIFF)

Text S1 **Supplementary text analysing in more detail several points in the manuscript.** To be read in conjuction with the accompanying supplementary figures.
(PDF)

Text S2 **The MATLAB/C99 source code we used in this study.**
(BZ2)
