# Supplementary figures and images for: A Self-Organizing State-Space-Model Approach for Parameter Estimation in Hodgkin-Huxley-Type Models of Single Neurons
Source: PLoS Comput Biol. 2012 Mar 1;8(3):e1002401. doi: 10.1371/journal.pcbi.1002401 (PMC3291554; doi:10.1371/journal.pcbi.1002401)

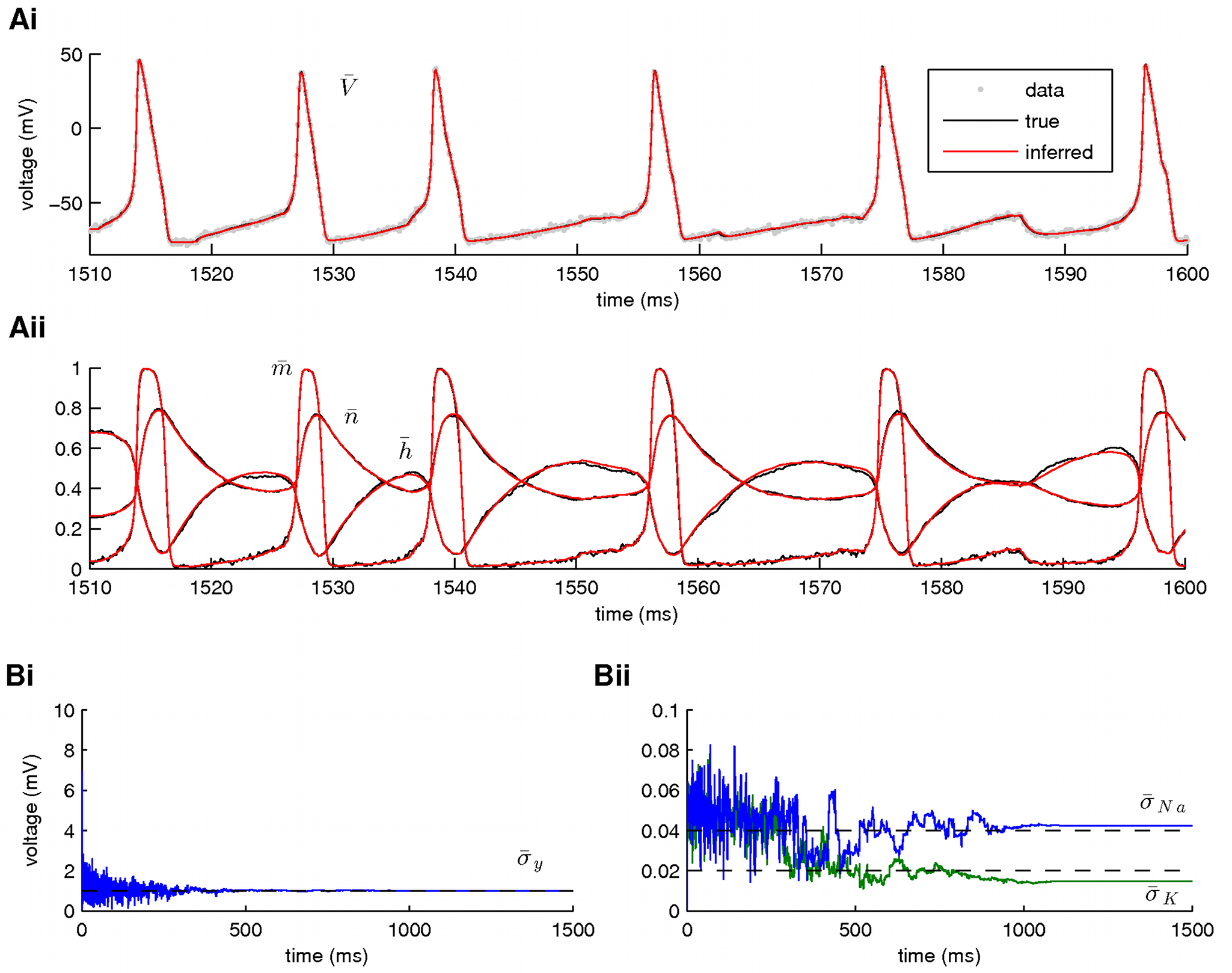

Supplement: Figure S1 — Simultaneous estimation of hidden states and channel noise in a stochastic single-compartment model. Estimation was based on a simulated -long recording of membrane potential generated by Supplementary Eqs. S1, S2 and S4. For clarity, only of activity are shown in Figs. Ai,ii. Notice that in these simulations, we assumed the absence of synaptic input (i.e. ). Activity in the model neuron was driven by a random sequence of current steps with amplitude between and and duration up to . (A) Simultaneous inference of the observed membrane potential (Ai) and the hidden activation (, ) and inactivation () gating variables for the sodium and potassium currents. (B) Inference of the standard deviation of the observation noise (Bi) and the parameters and , which control the variance of the sodium and potassium channel noise (Bii). Estimates converged to their final values after approximately . The dashed lines indicate the true values of these parameters. The y-axes in Bi,ii indicate the width of the prior intervals imposed on the corresponding parameters. Simulation parameters were: , and . The prior interval for the scaling factors was . (TIFF) [file pcbi.1002401.s001.tiff]

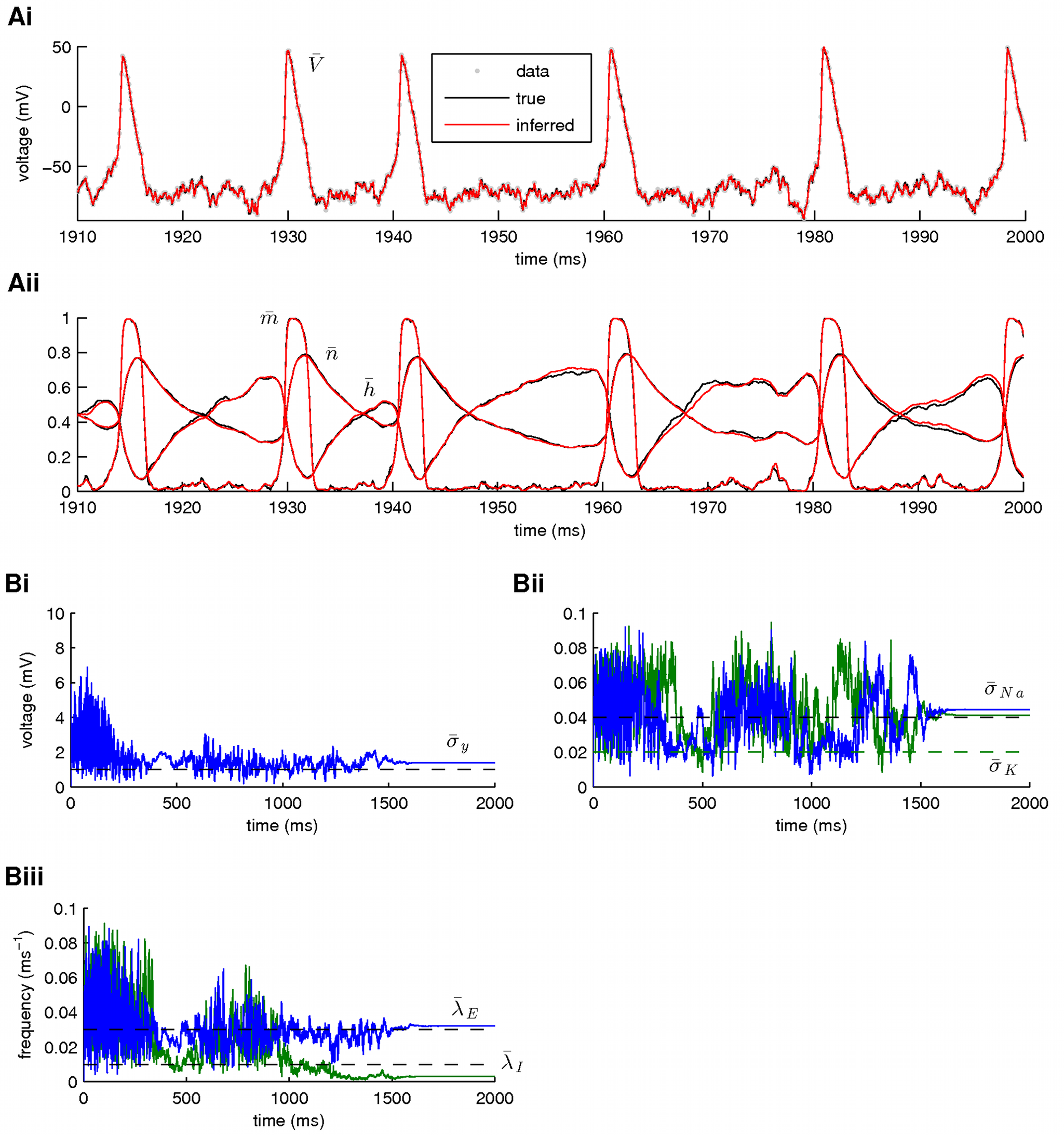

Supplement: Figure S2 — Simultaneous estimation of hidden states, channel noise and presynaptic firing rates in a stochastic single-compartment model. Estimation was based on a simulated -long recording of membrane potential generated by Eqs. S1, S2 and S4 with . For clarity, only of activity are shown in Figs. Ai,ii. (A) Simultaneous inference of the observed membrane potential (Ai) and the hidden activation (, ) and inactivation () gating variables for the sodium and potassium currents (Aii). (B) Inference of the standard deviation of the observation noise (Bi), parameters and , which control the variance of the sodium and potassium channel noise (Bii) and the presynaptic firing rates and (Biii). Estimates converged to their final values after approximately of activity. The dashed lines indicate the true values of the parameters. The y-axes in B and C indicate the width of the prior intervals imposed on the corresponding parameters. Discrepancies from the true values in B are due to the overlapping effects of different parameters controlling observation, channel and synaptic noise. Simulation parameters were: , and . The prior interval for the scaling factors was . (TIFF) [file pcbi.1002401.s002.tiff]

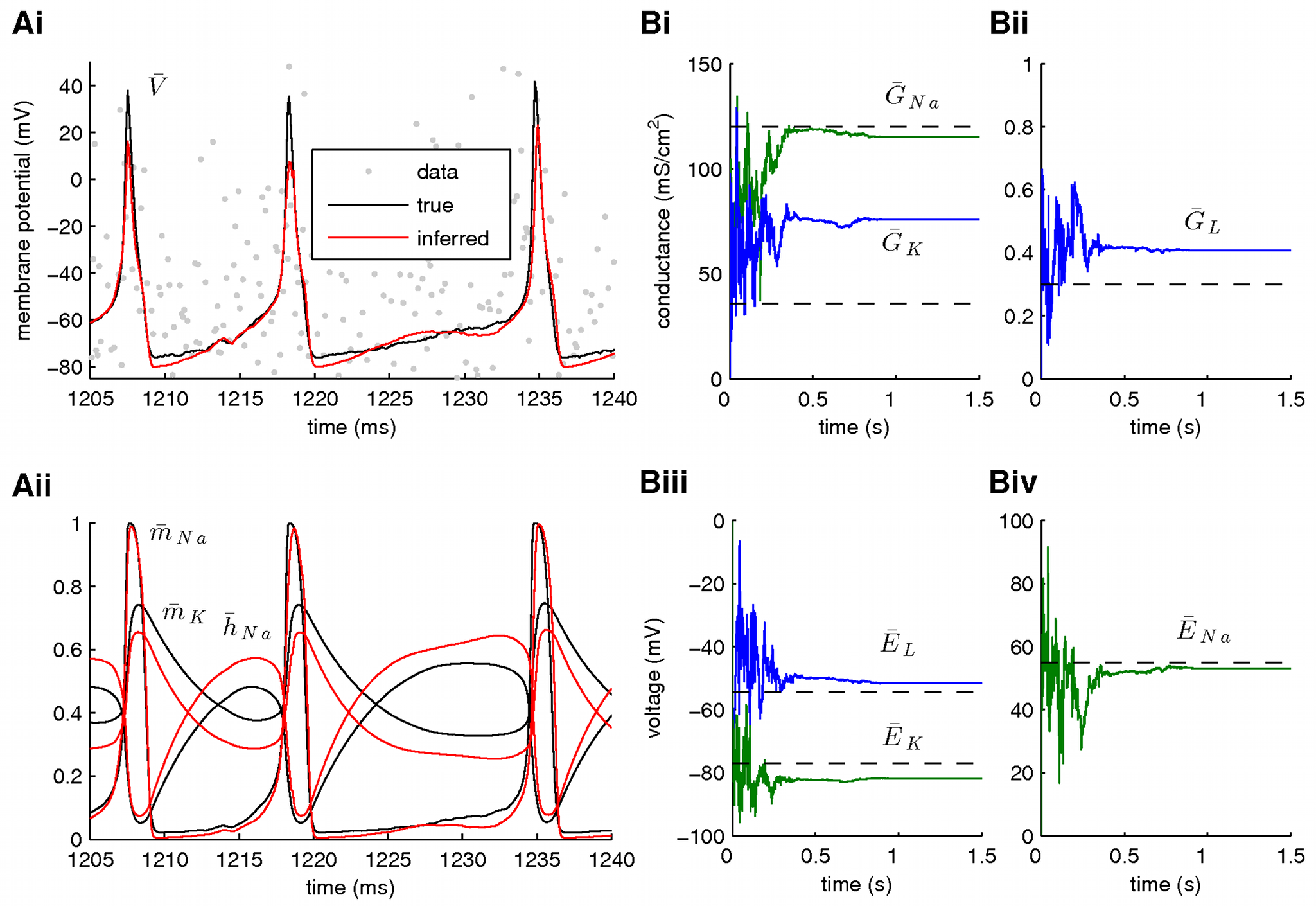

Supplement: Figure S3 — Simultaneous inference of hidden states and unknown parameters in the single compartment model (see main text) at high levels of observation noise. This figure corresponds to Fig. 7Dii in the main text for . (A) Inferred membrane potential (Ai) and unobserved gating variables (Aii). (B) Examples of simultaneously inferred parameters, such as maximal conductances (Bi,ii) and reversal potentials (Biii,iv). The y-axes in Bi–iv indicate the prior intervals of the corresponding parameters. Simulation details are as in Fig. 7 in the main text. (TIFF) [file pcbi.1002401.s003.tiff]

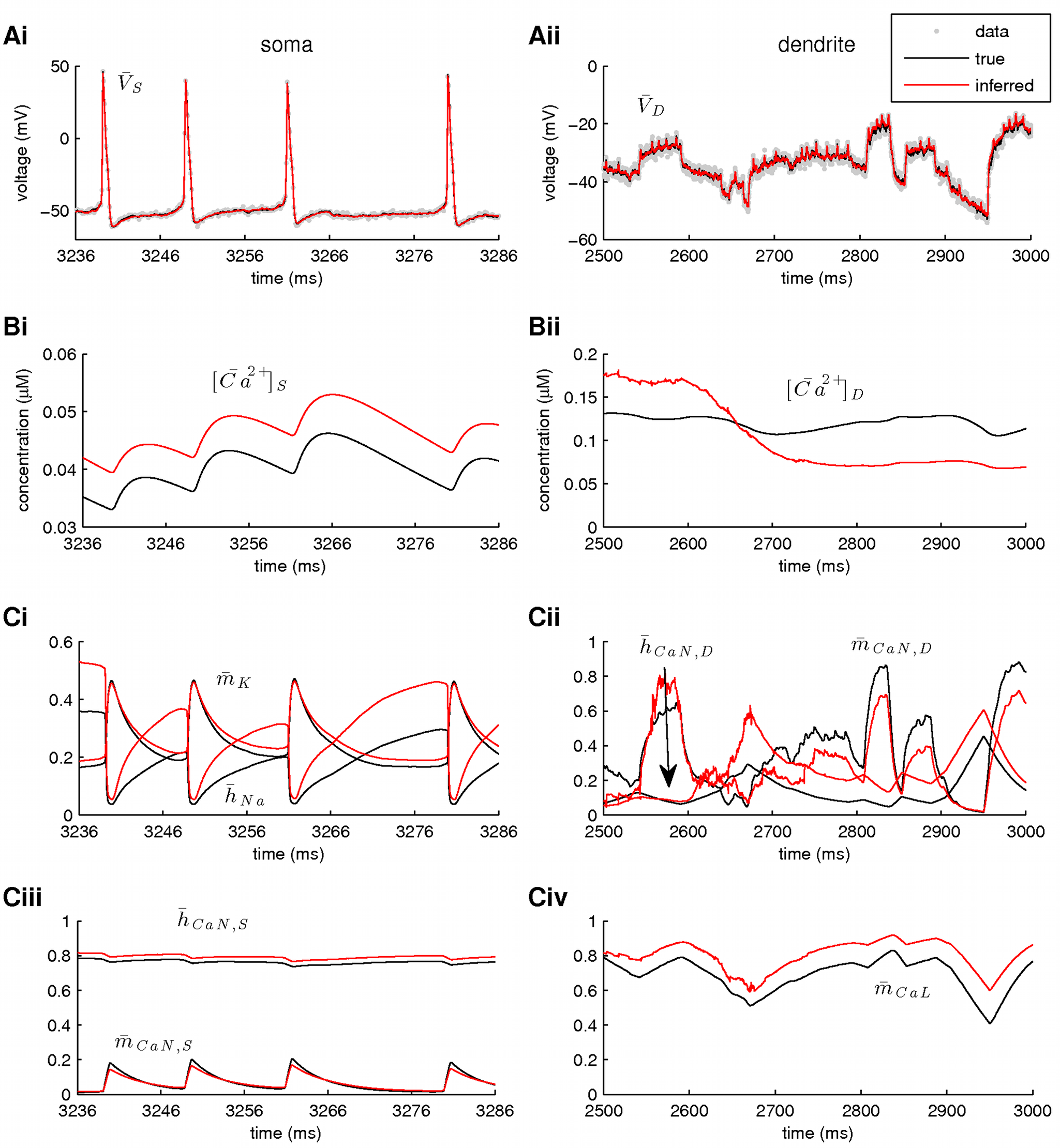

Supplement: Figure S4 — Simultaneous inference of hidden states in the two-compartment model (see main text) at low levels of observation noise. This figure corresponds to Figs. 11Aii for , 11B and 11C in the main text. (A) Inference of the membrane potential at the soma (Ai) and the dendritic compartment (Aii). (B) Inference of the unobserved concentration of intracellular calcium at the soma (Bi) and the dendritic compartment (Bii). (C) Inference of the unobserved gating variables for the sodium and potassium currents at the soma (Ci), the N-type calcium current at the soma (Ciii), the N-type calcium current at the dendritic compartment (Cii) and the L-type calcium current at the dendritic compartment (Civ). Simulation details are as in Fig. 11 in the main text. (TIFF) [file pcbi.1002401.s004.tiff]

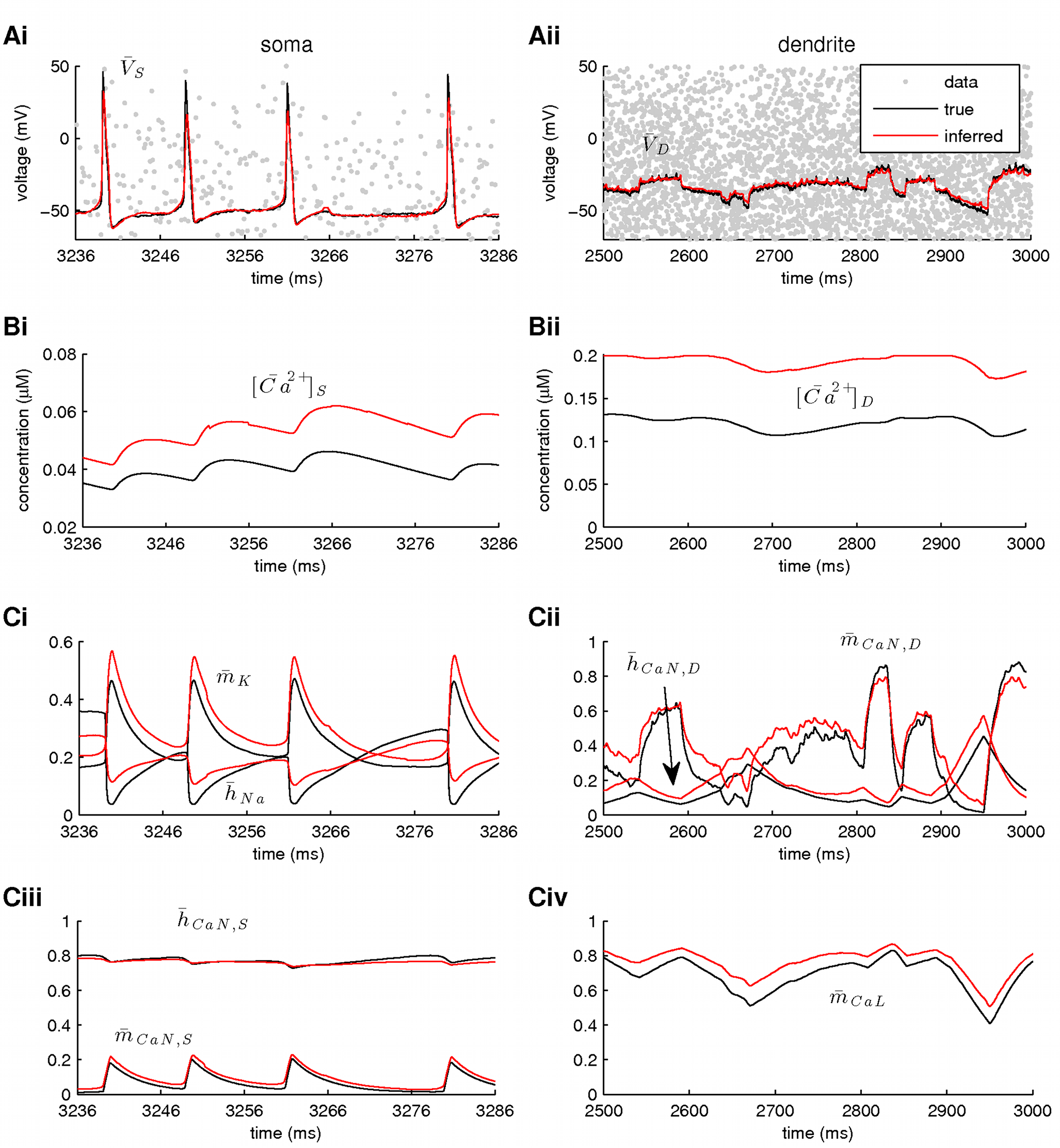

Supplement: Figure S5 — Simultaneous inference of hidden states in the two-compartment model (see main text) at high levels of observation noise. This figure corresponds to Fig. 11Aii for . (A) Inference of the membrane potential at the soma (Ai) and the dendritic compartment (Aii). (B) Inference of the unobserved concentration of intracellular calcium at the soma (Bi) and the dendritic compartment (Bii). (C) Inference of the unobserved gating variables for the sodium and potassium currents at the soma (Ci), the N-type calcium current at the soma (Ciii), the N-type calcium current at the dendritic compartment (Cii) and the L-type calcium current at the dendritic compartment (Civ). Simulation details are as in Fig. 11 in the main text. (TIFF) [file pcbi.1002401.s005.tiff]

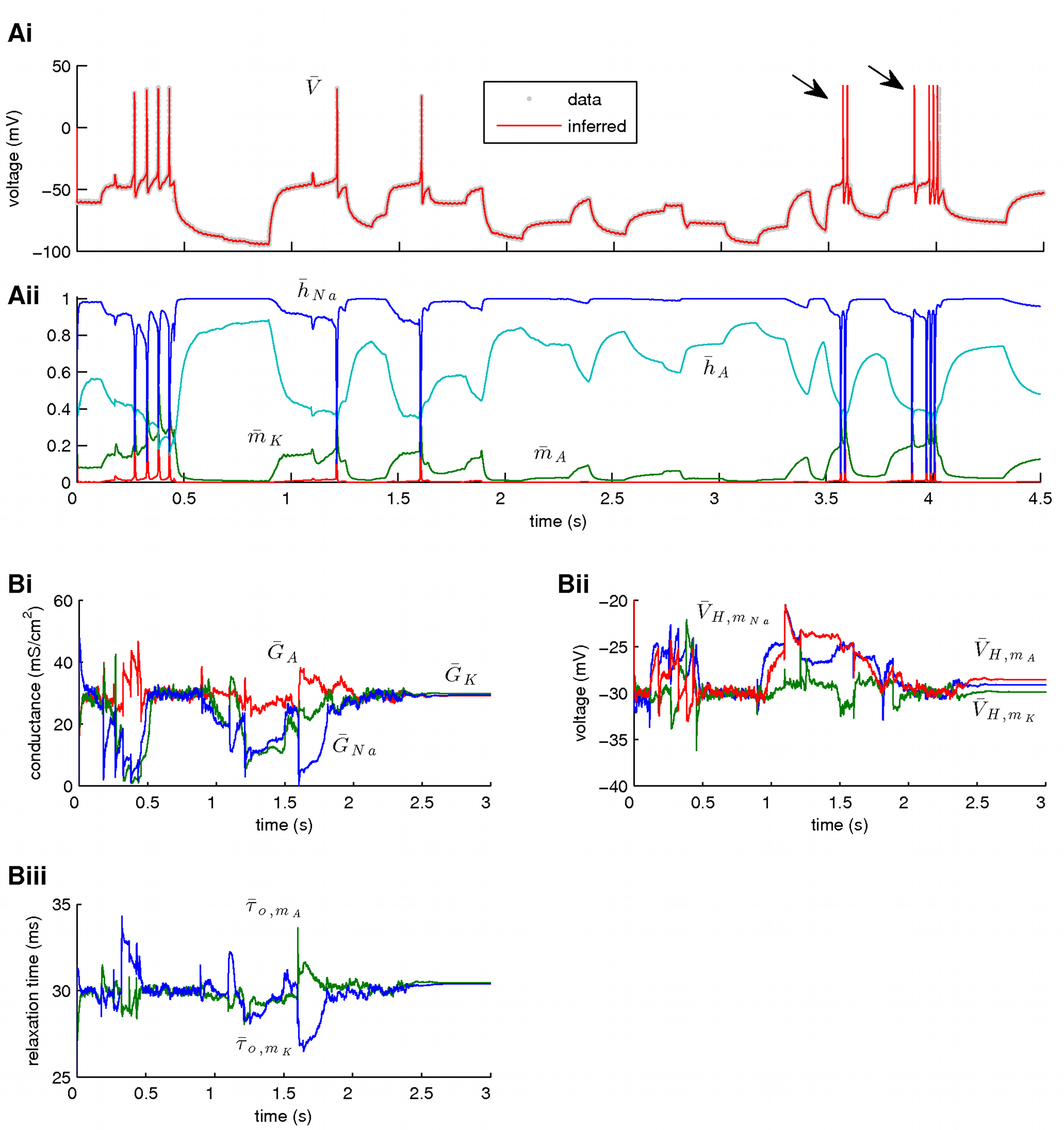

Supplement: Figure S6 — Inference in the B4 model using a single recording of the membrane potential. A single -long recording of B4 activity induced by injecting a sequence of random current steps in the neuron was used during smoothing. Random current amplitude was between and and random step duration was between and . (A) Inference of the membrane potential (Ai) and the unobserved gating variables for the sodium and potassium currents in the model (Aii). (B) Examples of simultaneously inferred model parameters: maximal conductances of all currents (Bi), half steady-state activation voltages for all currents (Bii) and maximal relaxation times for the activation of the potassium currents in the model (Biii). Notice that in all cases the parameter estimates converge exactly to the middle of their prior intervals (indicated by the y-axes in Bi–iii). This convergence takes place while the algorithm processes the “inactive” region of the data (approximately, from second to second 3 in Ai). Based on these converged estimates, the model incorrectly emits spikes later during smoothing (see arrows in Ai), indicating that the estimated parameters are not optimal for smoothing during the whole duration of experimental data. Simulation parameters were as follows: , and . The prior interval for the scaling factors was . For the free parameters in the model, we used the narrow prior intervals in Table 3. (TIFF) [file pcbi.1002401.s006.tiff]

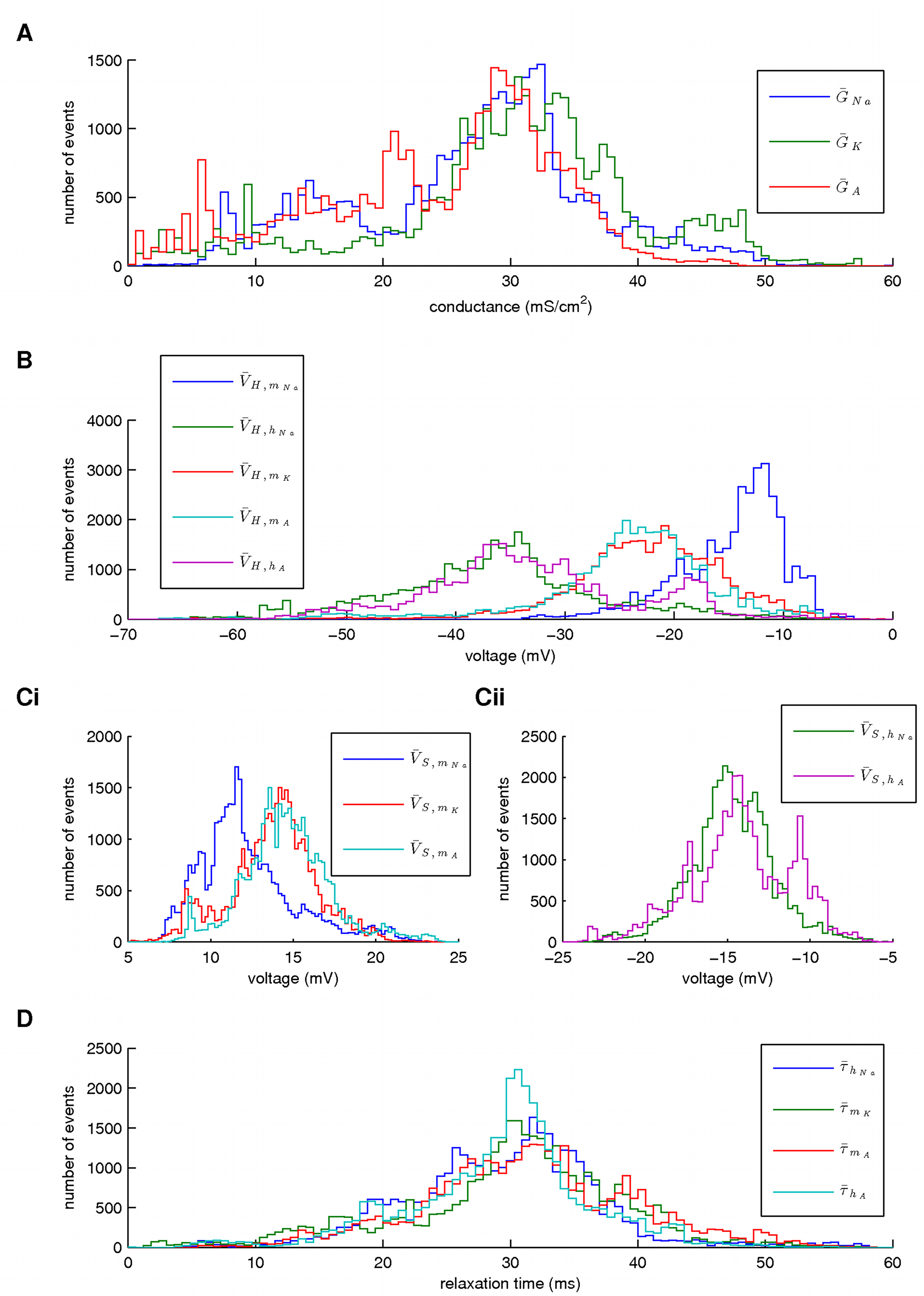

Supplement: Figure S7 — Inferred posterior distributions of all unknown parameters in the B4 model using the broad prior intervals in Table 3 . Inference was based on simultaneously smoothing four -long voltage recordings from the B4 neuron as in Fig. 12A in the main text. As in that case, data smoothing was accomplished with very high fidelity, as illustrated in Fig. 12A. (A) Inferred maximal conductances. (B) Inferred half steady-state activation and inactivation voltages. (C) Inferred activation (Ci) and inactivation (Cii) voltage sensitivities (parameters in the model). (D) Activation and inactivation relaxation times. The x-axes in all plots indicate the prior parameter intervals we used (Table 3). Notice that most posteriors are very broad (covering a large portion of the prior interval) and not unimodal. Simulation parameters were as described in Fig. 12 of the main text. (TIFF) [file pcbi.1002401.s007.tiff]
