# Supplementary material for: A Self-Organizing State-Space-Model Approach for Parameter Estimation in Hodgkin-Huxley-Type Models of Single Neurons
Source: PLoS Comput Biol. 2012 Mar 1;8(3):e1002401. doi: 10.1371/journal.pcbi.1002401 (PMC3291554; doi:10.1371/journal.pcbi.1002401)
Supplement: Text S1 — Supplementary text analysing in more detail several points in the manuscript. To be read in conjuction with the accompanying supplementary figures. (PDF) [file pcbi.1002401.s008.pdf]

A Self-Organizing State-Space-Model Approach for Parameter Estimation in Hodgkin-Huxley-Type Models of Single Neurons

Dimitrios V. Vavoulis[1,*], Volko A. Straub[2], John A. D. Aston[3], Jianfeng Feng[1,4,*]

1 Dept. of Computer Science, University of Warwick, Coventry, UK

2 Dept. of Cell Physiology and Pharmacology, University of Leicester, Leicester, UK

3 Dept. of Statistics, University of Warwick, Coventry, UK

4 Ctr. for Computational Systems Biology, Fudan University, Shanghai, PR China

∗ **E-mail: Dimitris.Vavoulis@dcs.warwick.ac.uk, Jianfeng.Feng@warwick.ac.uk**

Supplementary Material

There are several ways to introduce noise in the Hodgkin-Huxley-type neuron models as the ones we examined in this paper [1, 2]. A quite common approach is to add a white noise term in the right-hand side of the current conservation equation (which describes the evolution of the membrane potential in time), as seen for example in Eq. 31 in the main text. This "noisy current" aims to approximate the effect of a number of factors, such as the stochastic opening and shutting of transmembrane ion channels or the random bombardment of the neuron with synaptic input, and its major advantage is its simplicity. This is the approach we followed in this study. Since a major source of noise is the random fluctuations in the total conductance within a population of ion channels, it is reasonable to assume that similar (possibly, state-dependent) noise terms should be included in the dynamic equations describing the time evolution of the activation and inactivation gating variables (Eq. 32). For a single compartment model (as in Eqs. 31 and 32 in the main text), we can write:

$$dV = \frac{I_{ext} - G_L(V - E_L) - G_{Na}m^3h(V - E_{Na}) - G_K n^4(V - E_K)}{C_m} dt - \frac{1}{C_m} dI_{syn} \tag{S1}$$

$$dx = (a_x(1 - x) - b_x x)\, dt + \sigma_X \sqrt{a_x(1 - x) + b_x x} dW_x \tag{S2}$$

where $x \in \{m, h, n\}$, $X \in \{Na, K\}$ and $\sigma_X = \left(\sqrt{N_X}\right)^{-1}$ with N_X being the total number of sodium or potassium channels in the model. a_x and b_x are functions of voltage, as shown below:

$$
\begin{aligned}
a_m &= 0.1\frac{V+40}{1-\exp\left(-\frac{V+40}{10}\right)} \quad &,& \quad b_m = 4\exp\left(-\frac{V+65}{18}\right) \\
a_h &= 0.07\exp\left(-\frac{V+65}{20}\right) \quad &,& \quad b_h = \frac{1}{1+\exp\left(-\frac{V+35}{10}\right)} \\
a_n &= 0.01\frac{V+55}{1-\exp\left(-\frac{V+55}{10}\right)} \quad &,& \quad b_n = 0.125\exp\left(-\frac{V+65}{80}\right)
\end{aligned}
$$

Notice that the noise terms in Eq. S2 depend on both the voltage and the gating variables. Also notice that, in Eq. S1, I_{syn} is the sum of the excitatory and inhibitory synaptic input the neuron receives. For an infinitesimal change in this current, we can write:

$$dI_{syn} = \gamma_E(V - E_E)dP_E + \gamma_I(V - E_I)dP_I \tag{S3}$$

where dP_E and dP_I are Poisson processes, which model the random arrival of presynaptic excitatory and inhibitory spikes at firing rates λ_E and λ_I, respectively. γ_E and γ_I are unitary increases in the synaptic conductance and E_E and E_I are the reversal potentials of the excitatory and inhibitory synaptic currents, respectively. Assuming that the neuron receives a high-frequency barrage of presynaptic spikes, it is common to re-write the above expression for synaptic current using the diffusion approximation [3]:

$$dI_{syn} = \left(\gamma_E\lambda_E(V - E_E) + \gamma_I\lambda_I(V - E_I)\right)dt + \sqrt{\lambda_E\gamma_E^2(V - E_E)^2 + \lambda_I\gamma_I^2(V - E_I)^2}dW_{syn} \tag{S4}$$

Notice that we have assumed that changes in the total synaptic current are instantaneous. This is just an approximation, since changes in synaptic conductances have characteristic rise and decay relaxation times (see, for example, [4,5]). Observation noise was as in Eq. 7 in the main text with $\sigma_y = 1mV$.

In Eq. S1, the membrane capacitance, maximal conductances and reversal potentials were as follows: $C_m = 1nF/cm^2$, $G_L = 0.3mS/cm^2$, $G_{Na} = 120mS/cm^2$, $G_K = 36mS/cm^2$, $E_L = -54.4mV$, $E_{Na} = 55mV$, $E_K = -77mV$, $E_E = 0mV$ and $E_I = -75mV$. In Eq. S2, $\sigma_{Na} = 0.04$ and $\sigma_K = 0.02$. Unitary synaptic conductances and presynaptic firing rates in Eq. S4 were: $\gamma_E = 1mS/cm^2$, $\gamma_I = 1mS/cm^2$, $\lambda_E = 0.03ms^{-1}$ and $\lambda_I = 0.01ms^{-1}$. With these parameters, the model in Eqs. S1, S2 and S4 was active in the absence of any external input I_{ext}. Given a recording of this activity, the fixed lag-smoother

can be used for retrieving the hidden states of the model and various parameters that control channel and synaptic noise (σ_{Na}, σ_K, λ_E and λ_I), as shown in Figs. S1 and S2. This simulation experiment demonstrates the applicability of the algorithm, when more complex noise models are considered.

References

1. Feng J, editor (2004) Computational Neuroscience: a comprehensive approach. CHAPMAN & HALL/CRC.

2. Goldwyn JH, Imennov NS, Famulare M, Shea-Brown E (2011) Stochastic differential equation models for ion channel noise in hodgkin-huxley neurons. Phys Rev E Stat Nonlin Soft Matter Phys 83: 041908.

3. Hanson FB, Tuckwell HC (1983) Diffusion approximations for neuronal activity including synaptic reversal potentials. J Theoret Neurobiol : 127–153.

4. Destexhe A, Rudolph M, Fellous JM, Sejnowski TJ (2001) Fluctuating synaptic conductances recreate in vivo-like activity in neocortical neurons. Neuroscience 107: 13-24.

5. Richardson MJE, Gerstner W (2005) Synaptic shot noise and conductance fluctuations affect the membrane voltage with equal significance. Neural Comput 17: 923-47.
